# CircDCAF8 promotes the progression of hepatocellular carcinoma through miR-217/NAP1L1 Axis, and induces angiogenesis and regorafenib resistance via exosome-mediated transfer

**DOI:** 10.1186/s12967-024-05233-4

**Published:** 2024-05-30

**Authors:** Jiahao Gong, Guoyong Han, Zhiqiang Chen, Yinqi Zhang, Bin Xu, Chao Xu, Wen Gao, Jindao Wu

**Affiliations:** 1 Hepatobiliary Center, The First Affiliated Hospital of Nanjing Medical University, Nanjing, Jiangsu Province China; 2 Key Laboratory of Liver Transplantation, Chinese Academy of Medical Sciences, NHC Key Laboratory of Hepatobiliary Cancers, Nanjing, Jiangsu Province China; 3 Affiliated Hospital of Qingdao University, Qingdao University, Qingdao, Shandong Province China; 4 Department of Oncology, The First Affiliated Hospital of Nanjing Medical University, 300 Guangzhou Road, Nanjing, 210029 China

## Abstract

**Background:**

Circular RNAs (circRNAs), which are a new type of single-stranded circular RNA, have significant involvement in progression of many diseases, including tumors. Currently, multiple circRNAs have been identified in hepatocellular carcinoma (HCC). Our study aims to investigate the function and mechanism of circDCAF8 in HCC.

**Methods:**

The expression of circDCAF8 (hsa_circ_0014879) in HCC and para-carcinoma tissue samples was determined using quantitative real-time polymerase chain reaction (qRT-PCR). The biological function of circDCAF8 in HCC was confirmed by experiments conducted both in vitro and in vivo. And the relationship between circDCAF8, miR-217 and NAP1L1 was predicted by database and verified using qRT-PCR, RNA-binding protein immunoprecipitation (RIP) and dual-luciferase reporter assays. Exosomes isolated from HCC cells were utilized to assess the connection of exosomal circDCAF8 with HCC angiogenesis and regorafenib resistance.

**Results:**

CircDCAF8 is upregulated in HCC tissues and cell lines, and is linked to an unfavourable prognosis for HCC patients. Functionally, circDCAF8 was proved to facilitate proliferation, migration, invasion and Epithelial-Mesenchymal Transformation (EMT) in HCC cells. Animal examinations also validated the tumor-promoting characteristics of circDCAF8 on HCC. Besides, exosomal circDCAF8 promoted angiogenesis in HUVECs. Mechanistically, circDCAF8 interacted with miR-217 and NAP1L1 was a downstream protein of miR-217. CircDCAF8 promoted NAP1L1 expression by sponging miR-217. In addition, exosomes may transfer circDCAF8 from regorafenib-resistant HCC cells to sensitive cells, where it would confer a resistant phenotype.

**Conclusion:**

CircDCAF8 facilitates HCC proliferation and metastasis via the miR-217/NAP1L1 axis. Meanwhile, circDCAF8 can promote angiogenesis and drive resistance to regorafenib, making it a viable therapeutic target for HCC patients.

**Supplementary Information:**

The online version contains supplementary material available at 10.1186/s12967-024-05233-4.

## Background

Primary liver cancer was diagnosed in almost 906,000 new cases worldwide, resulting in approximately 830,000 cancer-related deaths in 2020. Primary liver cancer ranks as the sixth most prevalent malignancy and the third highest contributor to cancer-related fatalities worldwide [1]. The most prevalent histopathology of primary liver cancer is hepatocellular carcinoma (HCC) [2]. China is responsible for more than half of the world’s cases of hepatitis B virus infection, which is a major cause of the disease [2]. There is still an unfavorable prognosis for patients with HCC even with the widespread use of a complete treatment approach centered on radical resection that includes locoregional therapies, systemic therapies, neoadjuvant therapies and transplantation in clinical practice [3, 4]. Consequently, it is crucial to explore the molecular mechanisms underlying HCC pathogenesis.

Circular RNAs (circRNAs) are a kind of RNA that generate covalent single-stranded loops through back-splicing without a 5’ cap or a 3’ Poly A tail, and can resist ribonuclease cleavage [5]. Due to its structural stability, high conservation and tissue-specific expression, circRNAs have the potential to be a significant biomarker for a number of cancers, including HCC. Classical mechanisms associated with circRNAs include miRNA sponge, RNA binding protein, translation, etc [6–9]. It has been documented that numerous circRNAs are connected to various distinct tumors, such as the development of gastric cancer is inhibited by circDIDO1 through the encoding DIDO1-529aa and the regulation of PRDX2 protein stability [10] and circGPRC5A promote colorectal cancer progression by stabilizing PPP1CA and inducing YAP dephosphorylation [11]. In HCC, circ_101555 has been reported to have a carcinogenic impact via miR-145-5p/CDCA3 axis [12]. Circ-LRIG3 interacts with EZH2 and STAT3 and promotes HCC progression through a positive feedback pathway [13]. Although many circRNAs have been investigated in HCC, their mechanism remains largely unclear.

Exosomes are disc-shaped, 30–100 nm-diameter vesicles encapsulated by a lipid bilayer secreted by cells. It plays a role in transporting bioactive compounds including DNAs, RNAs, lipids, and proteins between cells, regulating the intercellular microenvironment and immune system [14]. Exosomes have also been reported in studies to be essential for the progression of tumors [15, 16]. There have also been some reports demonstrated that circRNAs are abundant and stably expressed in exosomes and influence tumor progression, immune escape and drug resistance by transferred from cells to cells via exosomes [17–19]. It implies that exosomal circRNAs might be a useful target for therapy as well as a diagnostic biomarker.

In this study, we discovered that circDCAF8 was upregulated markedly in HCC tissues and cell lines, and that elevated circDCAF8 levels would be associated with a poor prognosis in HCC patients. Experiments conducted in vitro and in vivo confirmed that circDCAF8 has carcinogenic properties. And the downstream miR-217/NAP1L1 axis was explored. Moreover, we discovered circDCAF8 could be delivered by exosomes, which would promote HCC angiogenesis and conferred regorafenib-resistant phenotype. In conclusion, circDCAF8 appears to be a viable target for HCC therapy.

## Methods

### Patients and samples

The Ethics Committee of the First Affiliated Hospital of Nanjing Medical University has granted approval for this investigation. Before this study began, all patients signed written informed consent. All tissue samples were collected from HCC patients during operations at the First Affiliated Hospital of Nanjing Medical University. The patients did not receive any anti-tumor therapy before surgery and were pathologically diagnosed with primary hepatocellular carcinoma after surgery.

### Cell lines and culture conditions

Human HCC cell lines, including HCC-LM3, Huh-7, Hep-3B, Hep-G2, YY8103, SK-Hep1, MHCC-97 H, MHCC-97 L and normal human cell lines THLE-2 and HUVECs were purchased from the Chinese Academy of Sciences Cell Bank (CASCB, Shanghai, China). All cells were cultured in DMEM (Bio-Channel Nanjing, China) supplemented with 10% FBS (VivaCell, Shanghai, China) and 1% Penicillin-Streptomycin at 37 °C with 5% CO2.

### Cell transfection and generation of regorafenib-resistant cells

The sh-circDCAF8 vector was constructed by designing and synthesizing shRNA that targets human circDCAF8. The Lv-circDCAF8 vector was created by constructing lentiviral vectors that also include human circDCAF8. The lentiviral vectors mentioned above were designed by GenePharma (Shanghai, China). Target cells were transfected using lentiviral vectors, and the stable transfected cells were chosen using puromycin and confirmed with qRT-PCR. The shRNA target sequences are listed in Table S1, and the full sequence of circDCAF8 is listed in Table S2. The mimic and inhibitor of miR-217 and their negative controls were obtained from GenePharma (Shanghai, China). The cells were cultured in 6-well plates and transfected plasmid or inhibitor using Lipofectamine 2000 (Invitrogen, USA). Hep-G2 and Hep-3B cells were chosen to be induced regorafenib resistant HCC cells. Regorafenib was purchased from MCE (MedChemExpress, NJ, USA). Regorafenib resistant HCC cells were established by long-term exposure to regorafenib. Specifically, HCC cells were first treated with a modest dosage of regorafenib (0.625 µM) for 2 weeks and then the medium containing regorafenib was exchanged with fresh complete medium for an additional 2 weeks. Afterwards, the regorafenib dose was progressively raised while the culture pattern was maintained. This process continued until the regorafenib dose reached 10 µM, the maximum clinically tolerated dose, and the remaining cells were regorafenib-resistant HCC cells.

### RNA and gDNA extraction

Human tissues and cells were subjected to RNA extraction using the RNA Quick Purification Kit (YiShanbio, Shanghai, China) following the provided instructions. The gDNA of cells was isolated using Genomic DNA Isolation Kit (Vazyme, Nanjing, China).

### RT-PCR and qRT‑PCR

cDNA was synthesized by reverse transcription using HiScriptIIQ RT SuperMix for qPCR (Vazyme, Nanjing, China). qRT-PCR analysis was carried out using the SYBR Green PCR Kit (Vazyme, Nanjing, China) and ABI 7900 assay system (Applied Biosystems, CA, USA). GAPDH served as an internal control. Foldchange was calculated by 2^−ΔΔCt^. Table S3 contains a list of all primer sequence used in this investigation.

### Agarose gel electrophoresis

circDCAF8 divergent and convergent primers (CMBIO, Shanghai, China) were used to amplify the cDNA and gDNA products. 1% agarose gel was made using agarose, 1× Tris-acetate-EDTA buffer (Beyotime, Shanghai, China), and dyed with Goldview nucleic acid (Biosharp, Hefei, China). The previously obtained products were mixed with DNA loading buffer (Beyotime, Shanghai, China), then added to agarose gel and electrophoresed for 1.5 h at 100 V using 1× TAE as electrophoresis solution. The outcomes were observed under UV lamp.

### RNase R treatment

A total of 5 µg RNA was subjected to incubation with 3 U/µg RNase R (Epicentre Biotechnologies, Shanghai, China) for 15 min at 37℃. Subsequently, the RNA was purified using the RNeasy MinElute Cleaning Kit (Qiagen, Shanghai, China). After RNase R treatment, the results were identified by qRT-PCR.

### CCK-8 assay

2000 cells in a 200 µL cell solution were added into 96-well plates and cultured for various time periods, respectively. After 2 h of incubation with CCK-8 Kit (Biosharp, Hefei, China), cell viability is determined by the light absorption value at 450 nm that the enzyme-labeler records.

### Colony forming assay

400 cells were cultivated for two weeks on a 6-well plate. After that, the cells were fixed for 30 min with 4% paraformaldehyde, stained for two hours with crystal violet, and twice cleaned with PBS. Record the quantity of clones in each well.

### Edu assay

Logarithmic phase cells were planted in 24-well plates. 100 µL EdU solution (RiboBio, Guangzhou, China) was added to each well and then incubated for 2 h. Apollo dyeing solution was configured according to the instructions and 300 µL was added to each well. Hoechst reaction solution was dissolved in deionized water at 1:100, and 300 µL was added to each well. Images were taken with a fluorescence microscope (Zeiss, Jena, Germany).

### Transwell assay

The invasion and migration assays were conducted using transwell chambers (Corning, USA) pre-covered or uncovered with Matrigel (BD Biosciences, USA), respectively. In the upper chamber, 2 × 10^4^ cells dissolved in 200 µL of serum-free media were added, while in the lower well, 600 µL complete medium was added. Following 48 h of incubation, the invaded and migrated cells were fixed with 4% paraformaldehyde for 30 min and then stained with crystal violet for 2 h. After utilizing a cotton swab to remove the cells off the top membrane surface, an inverted microscope was used to record the images.

### Wound healing assay

The bottom surface of a 6-well plate was marked every 1 cm. Once the cells reached 90% confluency, a 200 µL pipette was used to draw a vertical line down the center of the plate. PBS was used to clear away the cell debris. Then cells were cultured using FBS-free medium. Microscopic images were taken at 0 h, 24 h and 48 h according to the different marker positions.

### Immunofluorescence

The 24-well plate was positioned with a slide at the bottom, and 5 × 10^4^ cells were planted to each well. After culture for 24 h, cells were fixed with 4% paraformaldehyde, permeabilized with 0.5% TritonX-100 and then blocked with immunol staining blocking buffer (Beyotime, Shanghai, China) for 30 min. The cells were next incubated with primary antibody overnight at 4℃. Next day, following 2 h of fluorescent secondary antibody (Beyotime, Shanghai, China) incubation, the nuclei were stained with DAPI (Beyotime, Shanghai, China) for 15 min. The pictures were captured under confocal laser scanning microscopy (Zeiss, Jena, Germany).

### Western blot

Proteins from HCC cell lines were extracted using RIPA (Beyotime, Shanghai, China) and PMSF (Beyotime, Shanghai, China) and then equal amounts of proteins were separated by 10% SDS-PAGE (Epizyme, Shanghai, China). The isolated proteins were then transferred onto PVDF membranes (Merck Millipore, Burlington, MA, USA). After sealing the immunoblots by QuickBlock™ Blocking Buffer (Beyotime, Shanghai, China) for 15 min. The primary antibodies were incubated with the membranes at 4℃ overnight. Next day, the membranes were cultivated for 2 h at room temperature using secondary antibodies. The immunoreactive bands were quantified using ECL Western Blotting Kit (Biosharp, Hefei, China) and Image Lab software (Bio-Rad, Hercules, CA, USA). The antibodies used in our research are displayed in Table S4.

### Xenograft nude mouse model

The Institutional Animal Care and Use Committee of the First Affiliated Hospital of Nanjing Medical University authorized the animal experiments used in this study (IACUC-2,208,013). The IACUC operational rules are followed for all animal-related operations. All animals used in this study were acquired from Vital River (Beijing, China). 24 male BLAB/C nude mice, aged 4–5 weeks, were allocated into 4 groups, with 6 mice in each group. Then each mouse’s left upper limb was injected under the armpit with 5 × 10^6^ lentivirus-transfected cells. For the regorafenib treatment assay, following the successful development of a subcutaneous tumor, regorafenib was given daily by intraperitoneal injection for 14 days. A 1:9 ratio of DMSO (Sigma, USA) to corn oil (MCE, USA) was used to suspend regorafenib. The regorafenib suspension was administered at a concentration of 50 mg/kg. 28 days after injection, subcutaneous tumor volumes were measured every 4d. After the mice were euthanized, the subcutaneous tumors were resected for weighing and immunohistochemical staining.

### Pulmonary metastasis model

Male BLAB/C nude mice (Vital River, Beijing, China) aged 4–5 weeks were divided into 4 groups with 6 mice per group. 1.5 × 10^6^ luciferase-expressing cells suspended in 100 µL PBS were intravenously in the tail vein into each mouse. After 4 weeks of cell injection, an IVIS Spectrum live imaging system (PerkinElmer, USA) was used to track the progress and metastasis of the tumor. Then the lungs were photographed and stained with HE after the mice were euthanized.

### H&E staining

Following their fixation in 10% formalin, tissues were processed and paraffin-embedded. Hematoxylin and eosin staining was applied to the 10 μm thick slices for morphological observation.

### Exosome isolation

Cells were cultured in 150 mm dishes. When the cell fusion rate reached more than 50%, the DMEM containing 10% exosome-free FBS was replaced, and the supernatant was collected after continued culture for 48 h. Exosomes were isolated by gradient centrifugation. In short, the collected cell supernatant was transferred into centrifuge tubes and centrifuged at 500 g for 10 min, 2000 g for 20 min, and 10,000 g for 30 min. The supernatant was collected after each centrifugation and transferred to a new centrifuge tube. After the third centrifugation, the supernatant is reserved and deposited in Beckmann tubes (Beckman, Brea, CA, USA). After that, the samples were ultra-centrifuged twice for 70 min at 110,000 g each time. The supernatant was abandoned and the precipitated exosomes were suspended in PBS.

### Exosome identification

The isolated exosomes were validated using a Transmission Electron Microscope (TEM). Nanoparticle Tracking Analysis (NTA) was used to measure the concentration and particle size distribution range of exosomes. Proteins were isolated from these exosomes, and western blotting was used to identify the exosome-related proteins: TSG101, HSP70 and CD63.

### Exosome uptake

The exosome suspension was supplemented with 100 µL of pre-made working solution PKH67 (War bio, Nanjing, China). After 30 min of room temperature incubation, the samples were mixed with PBS solution containing 5% BSA to end the incubation process. Cells were co-cultured with PKH67-labeled exosomes for 12 h. Following membrane permeabilized by 0.5% TritonX-100, the nucleus was stained with DAPI and photographed using laser confocal microscopy (Zeiss, Jena, Germany).

### Tubule formation

Each well of the 24-well plate was filled with 200 µL of Matrigel matrix (BD Biosciences, Franklin Lakes, NJ, USA) and the Matrigel matrix was then allowed to completely solidify on the plate by incubating the plate at 37 °C for 30 to 60 min. 1 × 10^5^ HUVEC cells suspended in 500 µL exosome-free medium were planted into each well. The formation of HUVEC cell tubules were examined under a light microscope after 6–8 h, and photos of the experimental outcomes were taken.

### RNA-binding protein immunoprecipitation (RIP)

Immunoprecipitations were performed according to the Magna RIP RNA-Binding Protein Immunoprecipitation Kit instructions (Millipore, MA, USA). AGO2 antibody (Cell Signaling Technology, Beverly, MA) was used for RIP. The Co-precipitated RNAs were subjected to RT-qPCR analysis.

### Dual-luciferase reporter assay

The circDCAF8-WT, circDCAF8-MUT, NAP1L1-WT, NAP1L1-MUT were cloned into pGL3-basic vector (GenePharma, Shanghai, China). The WT or MUT vector was co-transfected into HEK-293T cells with miRNA mimics using Lipofectamine 3000. The dual luciferase reporting system (Promega) was used to measure the luciferase activity after 48 h of incubation.

### Statistical analysis

Student’s t-test was used for comparison between the two groups, and one-way ANOVA was used for the multi-group comparison experiment. The relationships between circDCAF8 expression and clinicopathological characteristics of HCC patients were calculated by χ2 test. Spearman correlation was used to assess correlations. Kaplan-Meier technique was used to create survival curves. Statistical analyses were performed using GraphPad Prism 9.0. Data was reported by means ± SD. *P* < 0.05 was considered statistically significant.

## Results

### CircDCAF8 was identified as the potential circRNA in HCC

**Fig. 1:**
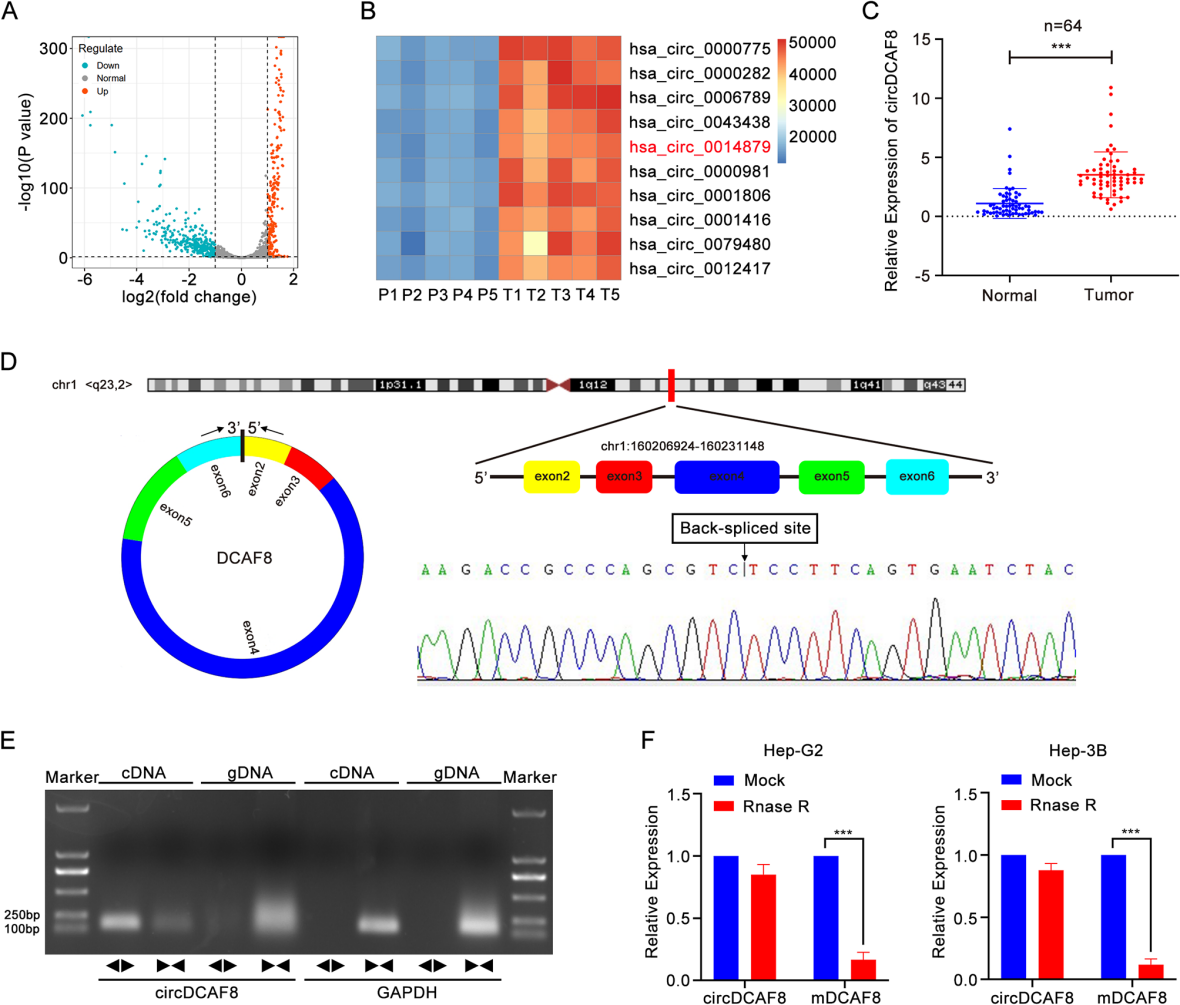
Selection and identification of circDCAF8. **A** The volcano plot of DECs in GSE94508. **B** A heatmap of the top 10 upregulated circRNAs in 5 paired samples of HCC. **C** Relative expression of circDCAF8 in human HCC tissues and paired adjacent nontumor tissues of 64 patients was determined by qRT-PCR. **D** Sanger sequencing of the annotated genomic region of circDCAF8 was performed to confirm the Back-spliced site of circDCAF8. **E** The divergent primers detected circDCAF8 in cDNA but not in gDNA by agarose gel electrophoresis. GAPDH was used as a negative control. **F** qRT–PCR analysis for the expression of circDCAF8 and mDCAF8 after treatment with RNase R in Hep-G2 and Hep-3B cells. Data are representative of three independent experiments and are presented as means ± SDs. (*p < 0.05; **p < 0.01; ***p < 0.001)

To discover the potential circRNAs associated with HCC, we conducted a search in the GEO database. Specifically, GSE94508 contained 2572 circRNAs detected in 5 paired samples of HCC and matched para-cancerous tissues was utilized for our analysis, of which 341 circRNAs were downregulated and 262 circRNAs were upregulated (|fold change|≥1.0 and padj ≤ 0.05)(Fig. 1A). After the top 3 upregulated circRNAs with the highest foldchange due to abnormal individual values were excluded (hsa_circ_0008661, hsa_circ_0050867 and hsa_circ_0004519), we chose the rest top 10 upregulated circRNAs (Fig. 1B) and examined these circRNAs in 16 human HCC tissue samples and matched para-cancerous tissues by qRT-PCR. Results showed 9 circRNAs could be amplified in HCC and 6 of them were statistically significant. CircDCAF8(hsa_circ_0014879) was ultimately selected for our investigation because its P value in the qRT-PCR is the most significant (Fig. S1).

We further validated that the level of circDCAF8 expression was significantly elevated in 64 paired HCC tissues and matched para-cancerous tissues (Fig. 1C). Additionally, a cohort of 64 HCC patients was divided into two groups according to the median expression of circDCAF8 in order to better investigate the clinicopathological characteristics of circDCAF8 in HCC (Table 1). The results revealed high circDCAF8 level was more likely to be related to large tumor diameter and advanced TNM stage (*P*<0.05, Chi-square test) while no significant difference in other clinical characteristics was detected (*P*>0.05). Collectively, our results demonstrated that circDCAF8 was increased in HCC and it could be a potential biomarker for HCC.

CircDCAF8 is derived from chr1:160206924–160,231,148 and composed of 5 consecutive exons within the DCAF8 gene. Sanger sequencing was performed to validate the specific back-splicing junction sequence in order to explore whether circDCAF8 was circular (Fig. 1D). To further identify the ring structure, we also designed convergence and divergence primers, and the amplification of circDCAF8 using convergent primers was seen in cDNA and gDNA samples, while the amplification of circDCAF8 using divergent primers was only found in the cDNA sample according to PCR results assessed by agarose gel electrophoresis (Fig. 1E). Furthermore, after being treated with RNase R, it was shown that circDCAF8 was more stable than linear DCAF8, suggesting circDCAF8 possessed a closed loop structure (Fig. 1F).

**Table 1:** Correlation between circDCAF8 expression and clinicopathologic characteristics in HCC

Characteristics		Total	High group	Low group	*P* value
		64	*N* = 32	*N* = 32	
Age (years)	<60	23	10	13	0.434
	≥ 60	41	22	19	
Gender	Male	38	20	18	0.611
	Female	26	12	14	
HBsAg status	Positive	48	25	23	0.564
	Negative	16	7	9	
AFP (ng/ml)	<200	21	8	13	0.183
	≥ 200	43	24	19	
PIVKA-II(mAU/ml)	<40	25	11	14	0.442
	≥ 40	39	21	18	
Tumor multiplicity	Single	35	16	19	0.451
	Multiple	29	16	13	
Tumor diameter (cm)	≤ 5	26	9	17	0.042^*^
	>5	38	23	15	
TNM stage	I + II	28	10	18	0.044^*^
	III + IV	36	22	14	
Microvascular invasion	Negative	29	12	17	0.209
	Positive	35	20	15	

AFP, alpha fetoprotein; PIVKA-II, Protein Induced by Vitamin K Absence or Antagonist II; HBsAg, hepatitis B surface antigen

^*^P < 0.05

### CircDCAF8 promotes proliferation, migration, invasion and EMT in vitro

**Fig. 2:**
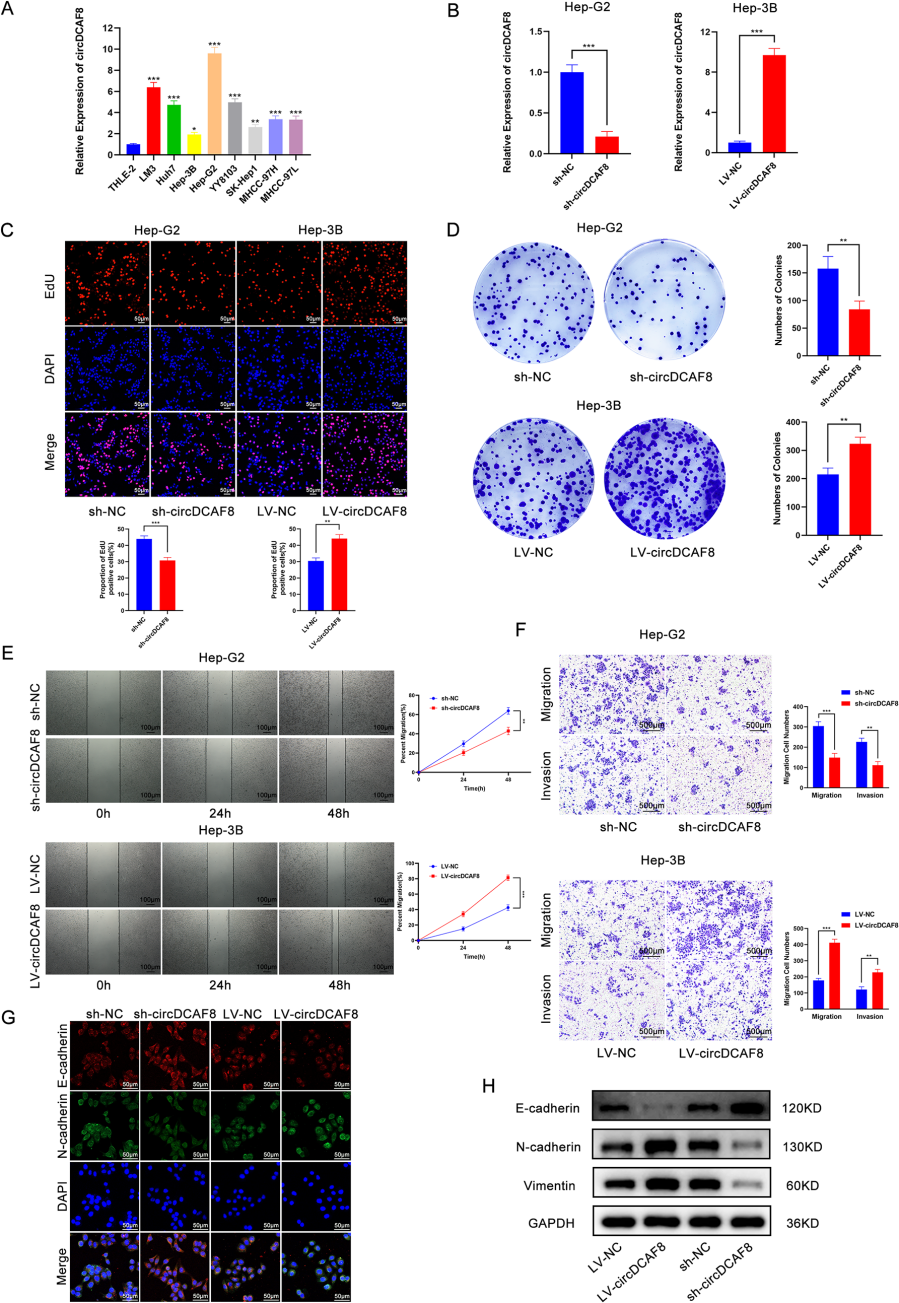
CircDCAF8 promotes proliferation, migration, invasion and EMT in HCC cells. **A** Relative expression of circDCAF8 in HCC cell lines and THLE-2 (human immortalized hepatocytes). **B** The knockdown efficiency of circCAF8 in Hep-G2 cells and overexpression efficiency in Hep-3B cells were determined by qRT-PCR. **C, D** EdU and colony formation assays evaluated the proliferation of sh-circDCAF8 cells and LV-circDCAF8 cells. Scale bar = 50 μm. **E** Wound healing assay determined cell migration ability. Scale bar = 100 μm. **F** Transwell assay measured invasion and migration ability with or without matrix. Scale bar = 500 μm. **G, H** Immunofluorescence and western blot detected the expression of EMT-related proteins. Scale bar = 50 μm. Data are representative of three independent experiments and are presented as means ± SDs. (*p < 0.05; **p < 0.01; ***p < 0.001)

To further explore the function of circDCAF8 in development of HCC, we conducted qRT-PCR to compare the expression of circDCAF8 in HCC cell lines to those in normal hepatocytes. The results indicated that Hep-G2 had the highest circDCAF8 expression, while Hep-3B had the lowest (Fig. 2A). As a result, in subsequent studies, we chose Hep-G2 to establish circDCAF8-stable knockdown cell lines through the infection of sh-circDCAF8, and Hep-3B to establish circDCAF8-stable overexpression cell lines using lentiviral vector infection. Stable cell lines for circDCAF8 knockdown and overexpression have been effectively established, as Fig. 2B illustrates. Using EdU and colony formation assays, the effects of circDCAF8 on HCC cell proliferation were examined. These findings demonstrated that suppressing circDCAF8 greatly inhibited the growth of Hep-G2 cells, whereas overexpressing circDCAF8 in Hep-3B cells led to the converse results (Fig. 2C, D). For the purpose of further investigating how circDCAF8 affects cell invasion and migration, we then carried out wound healing and transwell experiments. The outcomes demonstrated that circDCAF8 inactivation obviously decreased the migration and invasion capacity of Hep-G2, whereas circDCAF8 upregulation contributed to the opposite outcomes (Fig. 2E, F).

Epithelial-mesenchymal transformation (EMT) is a complicated procedure regulated by multiple factors, resulting in epithelial cells exhibiting mesenchymal characteristics linked to increased tumor metastasis, invasion, and chemotherapy resistance [20, 21]. In the process of EMT, Western blot (Fig. 2G) and immunofluorescence (Fig. 2H) experiments indicated that circDCAF8 knockdown led to an upregulation of E-cadherin and a downregulation of N-cadherin and Vimentin expression; while the expression of E-cadherin was downregulated and that of N-cadherin and Vimentin was upregulated upon circDCAF8 overexpression. These findings demonstrated circDCAF8 promoted EMT in HCC cells.

## CircDCAF8 promoted HCC proliferation and metastasis in vivo

**Fig. 3:**
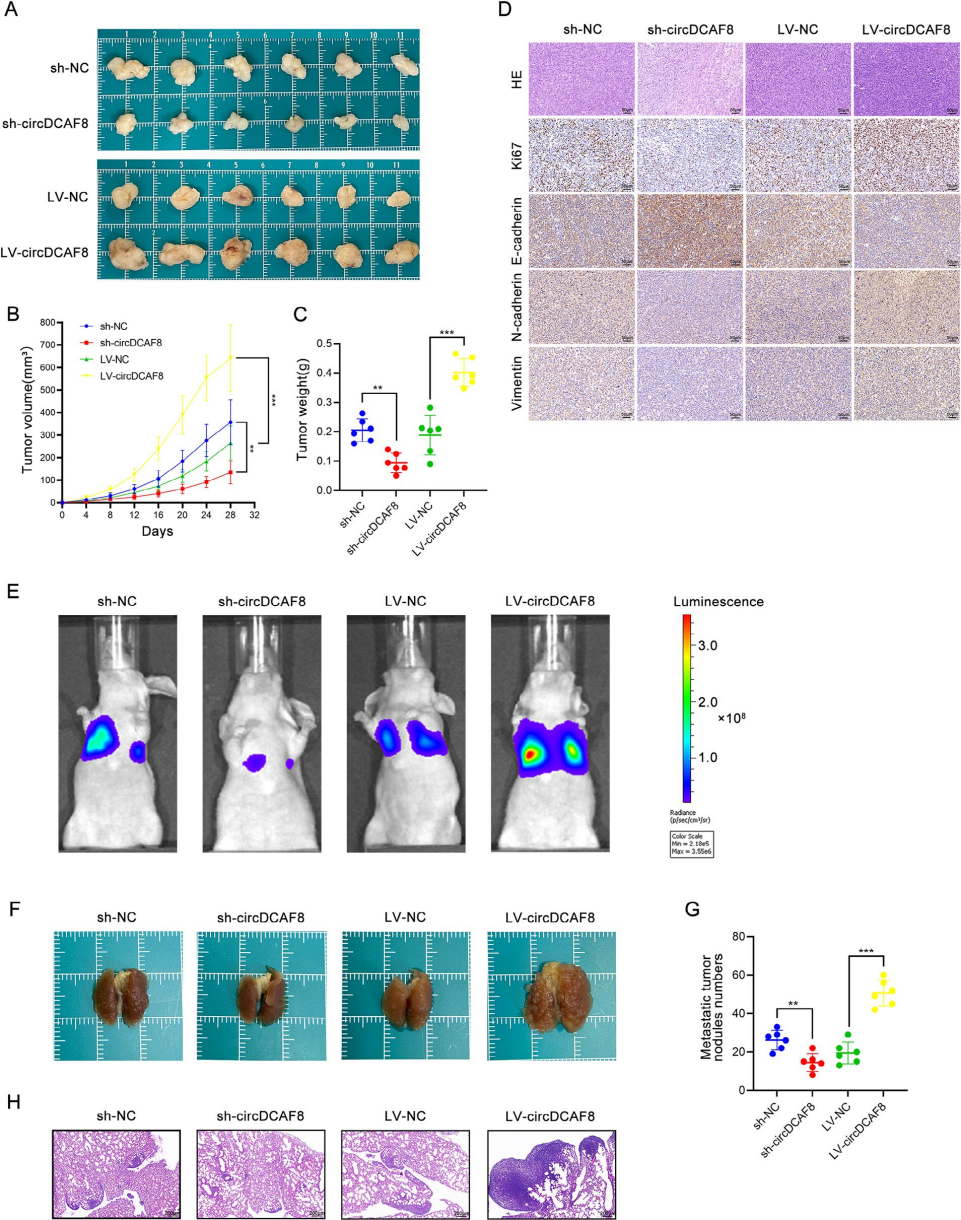
CircDCAF8 promoted HCC proliferation and metastasis in vivo. **A** Tumors formed in nude mice by subcutaneous injection of circDCAF8 stable knockdown or overexpression cells. **B, C** The volume and weight of the subcutaneous tumor. **D** H&E, Ki67, E-cadherin, N-cadherin and Vimentin staining of xenograft tumors. Scale bar = 50 μm. **E** Fluorescence intensity alterations in pulmonary metastasis models. **F** Lung metastasis induced by tail vein injection of circDCAF8 knockdown or overexpression cells in nude mice. **G** Lung metastatic nodules were counted. **H** H&E stain of lung metastases. Scale bar = 200 μm. *n* = 6 mice per group. Data are presented as means ± SDs. (*p < 0.05; **p < 0.01; ***p < 0.001)

To evaluate the impact of circDCAF8 on HCC proliferation and metastasis in vivo, we constructed tumor xenograft and lung metastasis models. According to the xenograft tumor models, tumor weights and sizes were both noticeably decreased in the sh-circDCAF8 group and considerably increased in the LV-circDCAF8 group (Fig. 3A-C). Additionally, the outcomes of immunohistochemical (IHC) staining also revealed that in the sh-circDCAF8 group, E-cadherin was upregulated while Ki67, N-cadherin, and Vimentin were downregulated. However, the LV-circDCAF8 group experienced the reverse outcome (Fig. 3D). Furthermore, in the lung metastasis models, 4 weeks after injection of the luciferase-expressing cells, low luminescence intensity was monitored in low circDCAF8 expression groups and the opposite result in high circDCAF8 expression groups (Fig. 3E). On lung specimens, less and smaller lung metastatic nodules were generated by circDCAF8 knockdown than circDCAF8 overexpression group (Fig. 3F-H). In conclusion, these results revealed that circDCAF8 facilitates proliferation and metastasis of HCC in vivo.

### CircDCAF8 transferred from HCC cells to HUVECs via exosomes and promoted angiogenesis of HCC

Through modifying the tumor microenvironment, tumor cells can stimulate angiogenesis, which in turn results in the early growth, invasion, postoperative recurrence, and metastasis of cancers [22, 23]. Thus, one of the key indicators of tumor aggressiveness is the capacity to generate angiogenesis. Exosomes are lipid bilayer microvesicles secreted by cells, which play a role in transferring active substances between cells as carriers [14]. Multiple investigations have shown that exosomes are essential for the development of tumors and circRNAs can be transmitted between cells via exosomes to regulate the tumor angiogenesis [17, 24, 25]. Therefore, we performed the following angiogenesis tests to investigate whether circDCAF8 promotes tumor angiogenesis through exosomes.

**Fig. 4:**
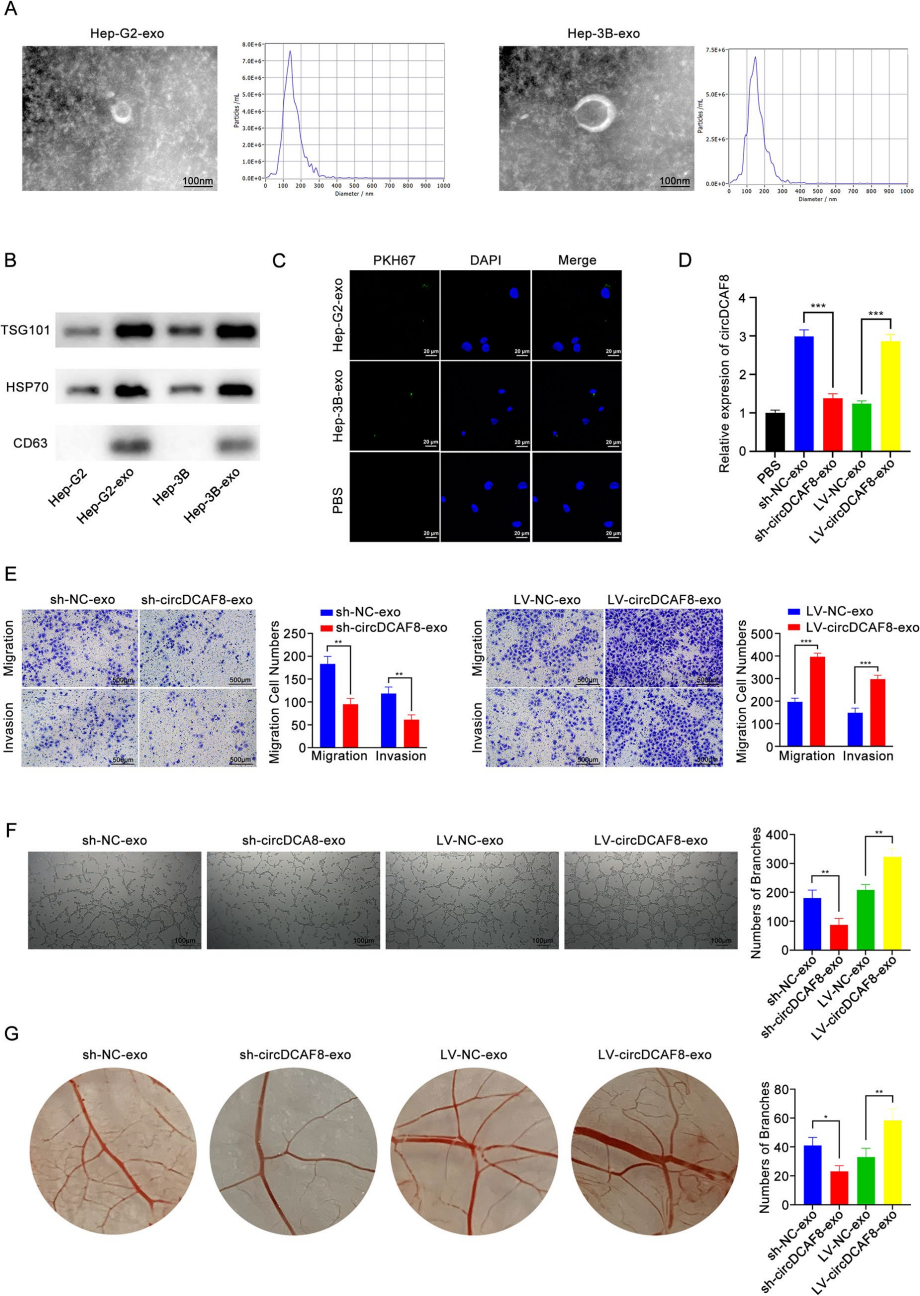
Exosomal circDCAF8 promoted the migration, invasion and tube formation of HUVECs. **A** TEM and NTA of exosomes isolated from Hep-G2 and Hep-3B. Scale bar = 100 nm. **B** Exosomal protein positive markers (Tsg101, HSP70 and CD63) were detected by western blot from purified exosomes and exosome-depleted cell extracts. **C** Laser confocal microscopy showed that the exosomes secreted by HCC cells were ingested by HUVECs. Scale bar = 20 μm. **D** qRT-PCR was performed to detect circDCAF8 expression in HUVECs after coculture with circDCAF8 knockdown or overexpression exosomes. **E** Migration and invasion of exosomes ingested by HUVECs was detected using the transwell assay. Scale bar = 500 μm. **F** Tube formation assay measured the tube formation ability of HUVECs ingested exosomes. Scale bar = 100 μm. **G** Chick chorioallantoic membrane assay showed that exosomal circDCAFB promoted the angiogenesis of chick embryo chorioallantoic membrane. Data are presented as means ± SDs. (*p < 0.05; **p < 0.01; ***p < 0.001)

In order to find out whether circDCAF8 can be transmitted by exosomes and contribute to angiogenesis, we started by isolating exosomes from Hep-G2 and Hep-3B cells with circDCAF8 knockdown or overexpression. Exosomes were isolated by differential centrifugation. Transmission electron microscopy (TEM) was used to identify the exosomes isolated from Hep-G2 and Hep-3B cells. Nanoparticle tracking analysis (NTA) confirmed that the average diameter of exosomes was 100 nm (Fig. 4A). The typical positive biomarkers of exosomes (Tsg101,HSP70 and CD63) were identified by WB (Fig. 4B).

Then we labeled exosomes by PKH67 and incubated with HUVECs for 24 h. Under confocal laser microscopy, a green fluorescence signal was observed in HUVECs (Fig. 4C), demonstrating that HUVECs successfully ingested the exosomes. qRT-PCR analysis revealed that the expression of circDCAF8 was downregulated in HUVECs incubated with sh-circDCAF8-exosomes and upregulated in HUVECs incubated with LV-circDCAF8-exosomes (Fig. 4D). After incubating for 24 h, exosomes isolated from circDCAF8 knockdown Hep-G2 cells reduced the proliferation of HUVECs, while those isolated from circDCAF8 overexpression Hep-3B cells led to the opposite results (Fig. S2A, B). Through the conduct of the transwell assay, it was discovered that sh-circDCAF8-exosomes decreased HUVECs’ capacity of migration and invasion whereas LV-circDCAF8-exosomes enhanced HUVECs’ capacity to do so when compared with control groups (Fig. 4E). In HUVECs tubule formation, the development of HUVECs tubules was inhibited by exosomes with circDCAF8 knockdown, but the opposite outcome was observed in those with circDCAF8 overexpression (Fig. 4F). In addition, the Chick Chorioallantoic Membrane (CAM) Assay revealed that exosomes exhibiting circDCAF8 downregulation reduced angiogenesis in the membrane and exosomes with upregulated circDCAF8 caused angiogenesis to rise (Fig. 4G). To sum up, these findings proved circDCAF8 could be transported by exosomes from HCC cells to HUVECs and stimulated HCC angiogenesis.

### CircDCAF8 acts as a sponge of miR-217

**Fig. 5:**
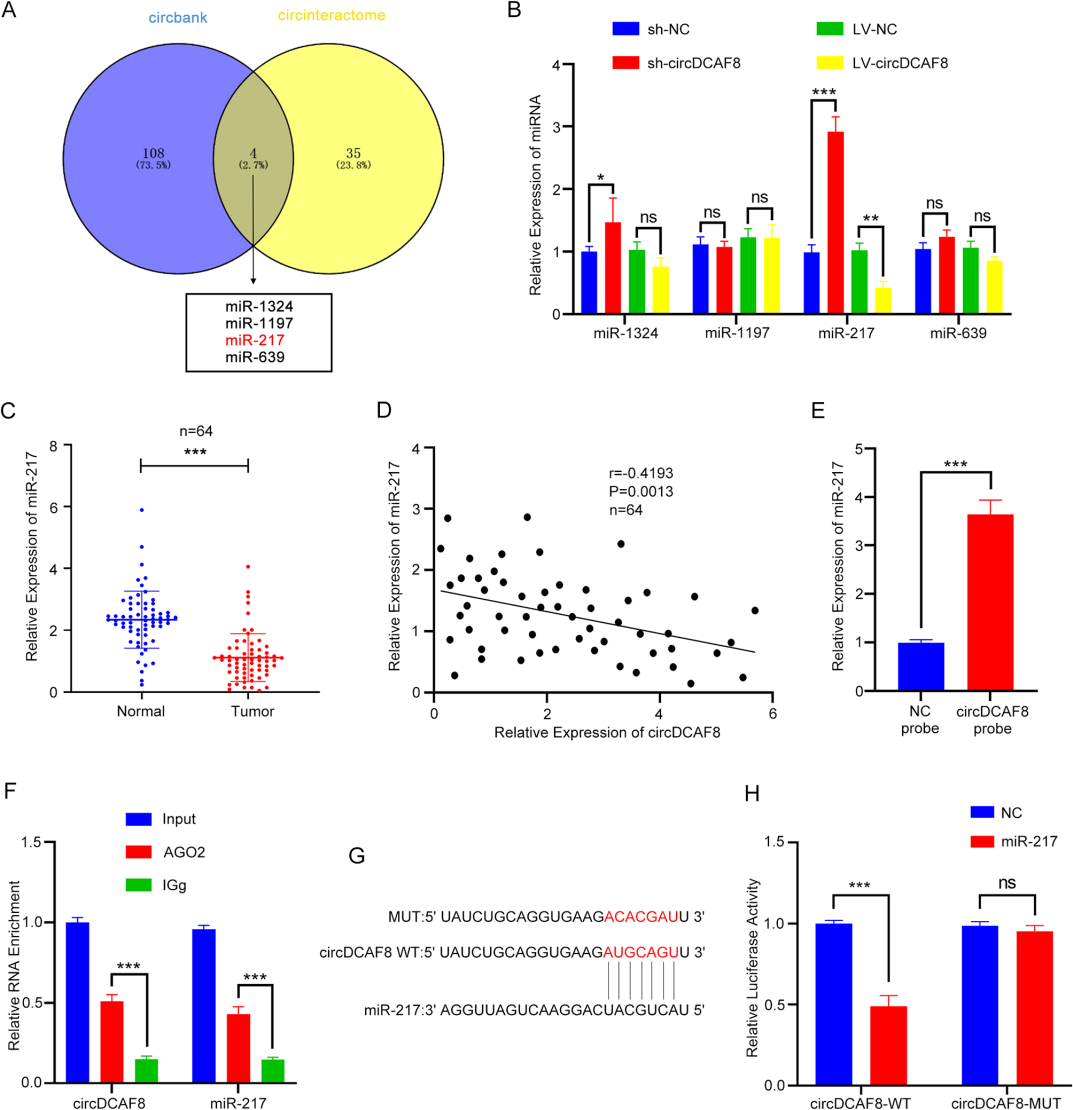
CircDCAF8 acts as a sponge of miR-217. **A** Downstream miRNAs of circDCAF8 predicted by circbank and circinteractome databases. **B** Relative expression of downstream miRNAs was determined by qRT-PCR in circDCAF8 knockdown and overexpression cells. **C** Relative expression of miR-217 in human HCC tissues and paired adjacent nontumor tissues of 64 patients was determined by qRT-PCR. **D** Spearman correlation analysis showed circDCAF8 expression was negatively correlated with the miR-217 expression. **E** Pull down assays showed that miR-217 was enriched by the circDCAF8 probe. **F** RIP assay confirmed circDCAF8 and miR-217 could bind with RNA-induced silencing complex (RISC). **G** A schematic of wild-type (WT) and mutant (MUT) circDCAF8 luciferase reporter vectors. **H** Luciferase reporter assay unveiled the molecular combination of miR-217 with circDCAF8 wild type in HEK-293T cells. Data are presented as means ± SDs. (ns, not significant; *p < 0.05; **p < 0.01; ***p < 0.001)

Existing researches have proved that circRNAs lead to tumor progression regularly by sponging specific miRNA [7, 8, 26]. We speculated that circDCAF8 may be a ceRNA that performs its biological function. Therefore, we used the circbank and circinteractome to investigate the possibility and ability of circDCAF8 to combine with miRNAs. MiR-1324, miR-1197, miR-217 and miR-639 were predicted to be the potential binding miRNAs of circDCAF8 (Fig. 5A). qRT-PCR results showed only miR-217 expression in HCC cells was markedly upregulated by circDCAF8 knockdown and downregulated by circDCAF8 overexpression (Fig. 5B). Subsequently, we confirmed that HCC tissues have lower levels of miR-217 than para-carcinoma tissues (Fig. 5C). Moreover, miR-217 expression and circDCAF8 showed a negative connection, according to correlation analysis (Fig. 5D). Accordingly, miR-217 was considered to be a downstream miRNA of circDCAF8. To further examine the interaction between circDCAF8 and miR-217, we conducted pull down assays using a biotin labeled circDCAF8 probe. As expected, the circDCAF8 probe enriched more miR-217 than NC probe (Fig. 5E). In addition, RIP assay confirmed that both circDCAF8 and miR-217 were found to enrich by the Ago2 antibody than IgG antibody control (Fig. 5F). We constructed wild-type (WT) and mutant (MUT) circDCAF8 vectors with luciferase based on the anticipated binding sites in order to further characterize circDCAF8 binding to miR-217 (Fig. 5G). Following co-transfection with miR-217 mimics, the luciferase intensity of WT vectors was much lower than that of MUT vectors (Fig. 5H). Combined with these results, it was proved that circDCAF8 acted as a sponge for miR-217.

### NAP1L1 is a target gene of miR-217

**Fig. 6:**
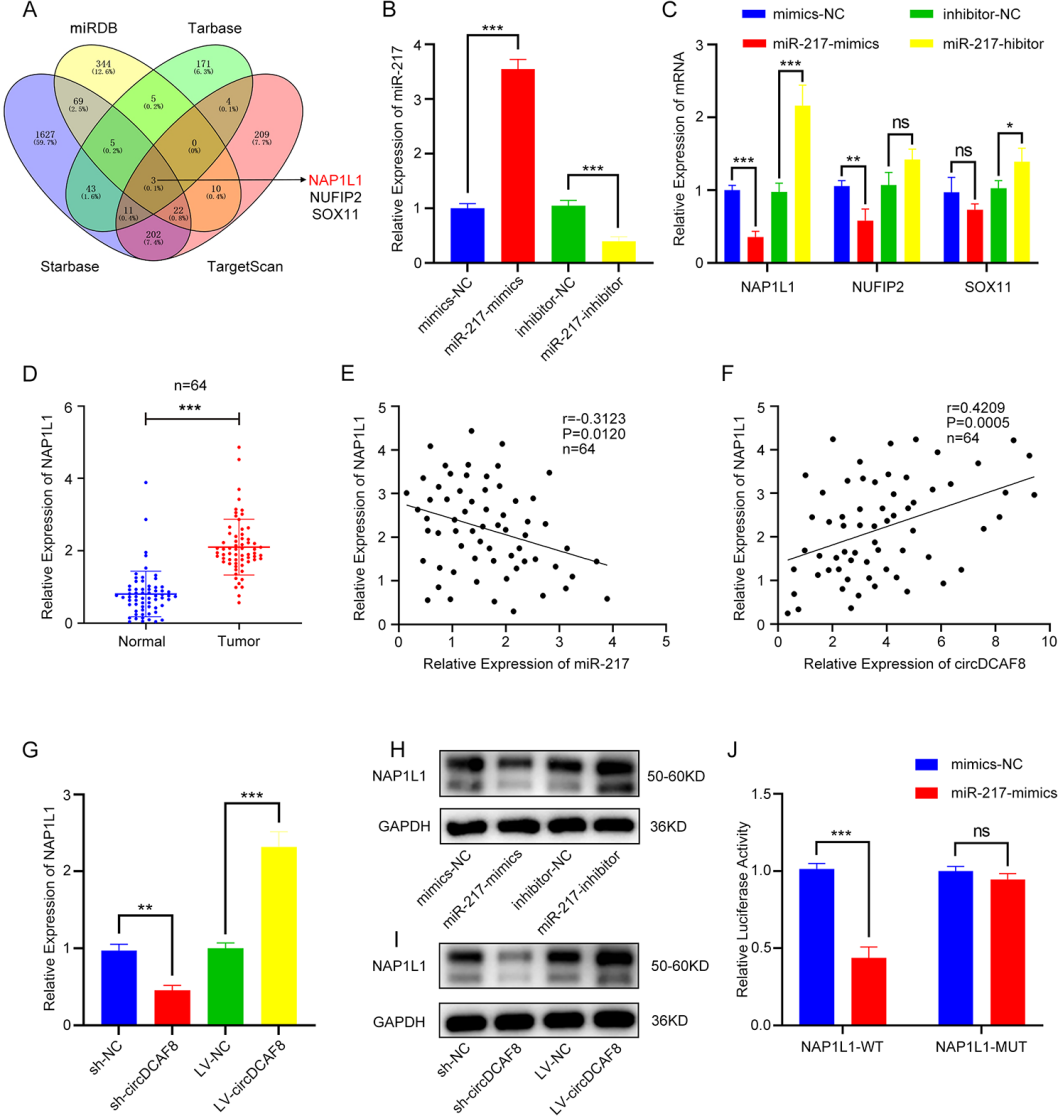
NAP1L1 is a target gene of miR-217. **A** Downstream genes of miR-217 predicted by miRDB, Tarbase, Starbase and TargetScan databases. **B** The overexpression and knockdown efficiency of miR-217 were determined by qRT-PCR. **C** Relative expression of downstream genes was determined by qRT-PCR in miR-217 overexpression and knockdown cells. **D** Relative expression of NAP1L1 in human HCC tissues and paired adjacent nontumor tissues of 64 patients was determined by qRT-PCR. **E, F** Spearman correlation analysis showed NAP1L1 expression was negatively correlated with the miR-217 expression(E) and positively correlated with circDCAF8 expression(F). **G** Relative expression of NAP1L1 was determined by qRT-PCR in circDCAF8 knockdown and overexpression cells. **H, I** Western blot was performed to detect protein expression levels of NAP1L1 in miR-217 and circDCAF8 knockdown or overexpression cells. **J** Luciferase reporter assay unveiled the molecular combination of miR-217 with circDCAF8 wild type in HEK-293T cells. Data are presented as means ± SDs. (ns, not significant; *p < 0.05; **p < 0.01; ***p < 0.001)

The miR-217 downstream target genes were predicted using the miRDB, Tarbase, Starbase, and TargetScan databases. Venn diagram cross-linking findings indicated that miR-217 altered the expression of several genes including NAP1L1, NUFIP2 and SOX11 (Fig. 6A). To investigate the interaction between miR-217 and the 3 candidate genes, we first constructed cell lines that overexpressed and knocked down miR-217 following the transfection of plasmid (Fig. 6B). qRT-PCR results demonstrated miR-217 mimics significantly suppressed NAP1L1 mRNA expression and miR-217 inhibitor markedly increased it (Fig. 6C). In additional, we performed qRT-PCR in HCC tissues and the outcomes demonstrated that only NAP1L1 was overexpressed compared with para-carcinoma tissue (Figs. 6D, S3A). NAP1L1 has been reported to be an oncogene in HCC [27, 28] and patients with low NAP1L1 had better overall survival (Fig. S3B). According to the spearman correlation analysis, NAP1L1 expression exhibited a negative connection with miR-217 (Fig. 6E) and a positive correlation with circDCAF8 (Fig. 6F). Meanwhile, in circDCAF8 overexpression cells, NAP1L1 mRNA expression was elevated, but in circDCAF8 knockdown cells it was downregulated as shown by qRT-PCR (Fig. 6G). Moreover, the findings from the WB confirmed that the expression of NAP1L1 was controlled by circDCAF8 and miR-217 (Fig. 6H, I). The binding sites of miR-217 were then validated using the dual-luciferase reporter assay, and the luciferase-containing NAP1L1-3′ UTR-WT and NAP1L1-3′ UTR-WT vectors were created in accordance with the Targetscan database (Fig. S3C). The luciferase reporter assay revealed that miR-217 mimics markedly reduced the luciferase activity of NAP1L1-WT while showing no discernible change in NAP1L1-MUT (Fig. 6J). Taken together, we concluded that miR-217’s target gene was NAP1L1.

### CircDCAF8 promoted HCC progression through the miR-217/NAP1L1 axis

**Fig. 7:**
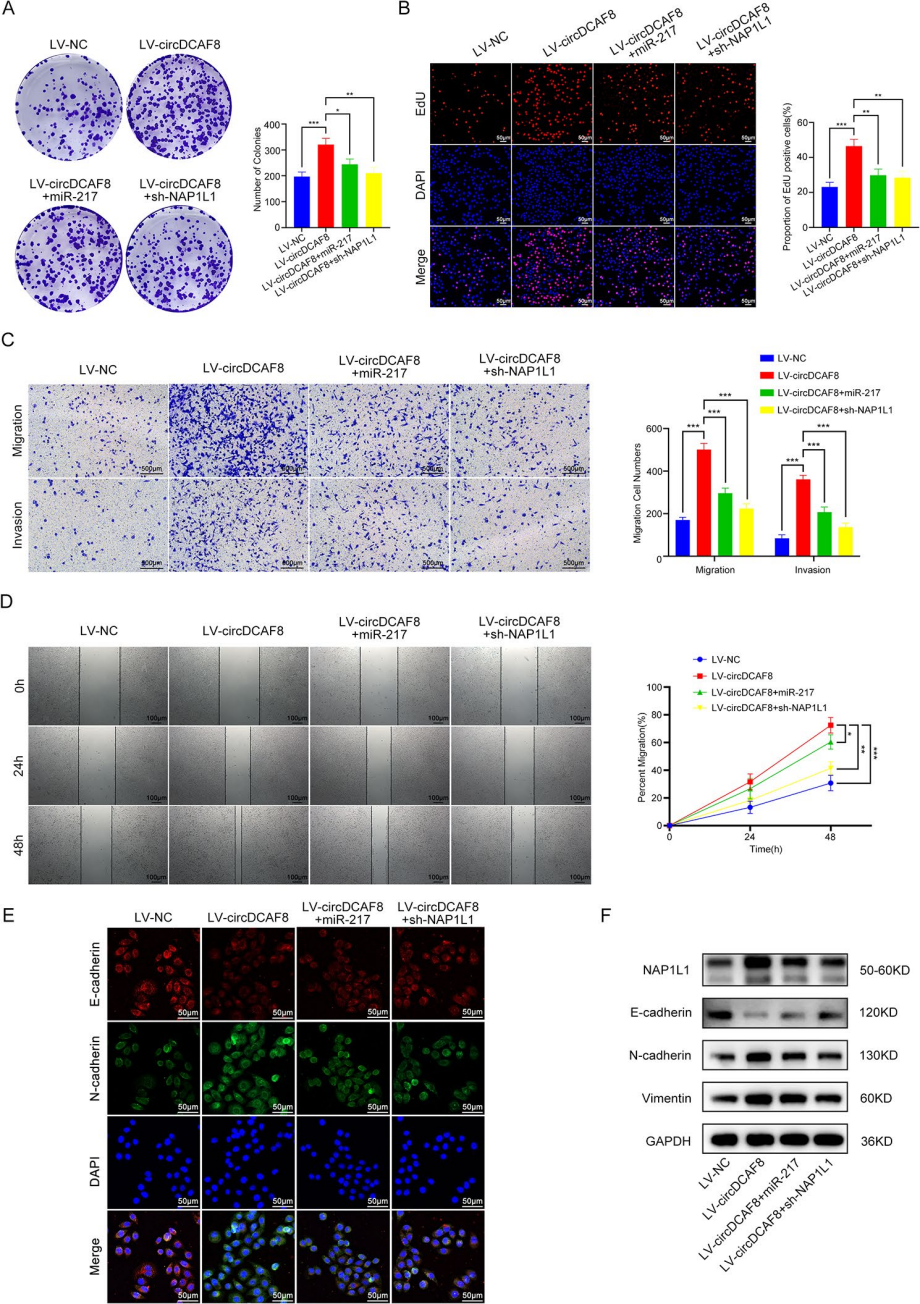
CircDCAF8 promoted HCC progression through the miR-217/NAP1L1 axis. **A, B** Colony formation and EdU assays evaluated the proliferation ability in LV-circDCAF8 Hep-3B cells and LV-circDCAF8 Hep-3B cells transfected with miR-217 mimics or sh-NAP1L1 vectors. Scale bar = 50 μm. **C** Transwell assay measured invasion and migration ability with or without matrix. Scale bar = 500 μm. **D** Wound healing assay determined cell migration ability. Scale bar = 100 μm. **E, F** Immunofluorescence and western blot detected the expression of NAP1L1 and EMT-related proteins. Scale bar = 50 μm. Data are representative of three independent experiments and are presented as means ± SDs. (*p < 0.05; **p < 0.01; ***p < 0.001)

To testify whether circDCAF8 accelerated the progression of HCC via the miR-217/NAP1L1 axis, we conducted rescue assays. MiR-217 mimics or sh-NAP1L1 vectors were co-transfected into circDCAF8 overexpression Hep-3B cells. As demonstrated by colony formation and EdU assay, miR-217 overexpression and NAP1L1 suppression counteracted the function of circDCAF8 overexpression on increasing cell proliferation (Fig. 7A, B). Transwell and wound healing assays revealed facilitation caused by circDCAF8 overexpression on cell migration and invasion were recovered by miR-217 overexpression and NAP1L1 suppression (Fig. 7C, D). In addition, LV-circDCAF8 downregulated the EMT-related protein E-cadherin; miR-217 mimics and NAP1L1 inhibitor also reversed the increase of N-cadherin and Vimentin (Fig. 7E, F), a quantification of the WB gels was displayed in Fig.S4A. In summary, circDCAF8 promotes HCC cell proliferation, migration, invasion and EMT by sponging miR-217 and upregulating the expression of NAP1L1.

To further investigate how NAP1L1 improves EMT in HCC, we used the HitpPedict and STRING databases to predict downstream target proteins (Fig. S4B). Among the 4 candidate proteins, UBE2O has been proved to bind to NAP1L1 [29] and promote EMT in head and neck squamous cell carcinoma [30] and breast cancer [31], but the relationship between UBE2O and NAP1L1 and EMT in HCC has not been studied. To verify whether NAP1L1 interacts with UBE2O in HCC, we performed immunofluorescence in Hep-G2 and Hep-3B. Laser confocal images confirmed the co-localization of NAP1L1 and UBE2O (Fig S4C). Immunoprecipitation and western blot assays in Hep-G2 and Hep-3B also demonstrated that UBE2O was co-immunoprecipitated by anti-NAP1L1 antibody, and NAP1L1 was also co-immunoprecipitated by anti-UBE2O antibody (Fig.S4D). These results suggested that NAP1L1 could bind to UBE2O in HCC. We next knocked down UBE2O in circDCAF8 overexpression and NAP1L1 overexpression Hep-3B cells to investigate the relationship between UBE2O and EMT. Western blot results demonstrated the inhibition of UBE2O increased the expression of E-cadherin and decreased the expression of N-cadherin and Vimentin. Meanwhile, the upregulation of N-cadherin and Vimentin and the decrease of E-cadherin caused by the overexpression of circDCAF8 and NAP1L1 could be reversed by UBE2O knockdown (Fig. S4E). Transwell assay was conducted to evaluate the effect of UBE2O on HCC cell migration and invasion. The findings demonstrated that UBE2O knockdown decreased the number of migrating cells and reversed the function of circDCAF8 and NAP1L1 overexpression on promoting cell migration and invasion (Fig. S4F). In conclusion, NAP1L1 can promote HCC EMT, migration and invasion by targeting UBE2O.

### CircDCAF8 is upregulated in regorafenib-resistant HCC cells and exosome-mediated circDCAF8 transfer and transmission of regorafenib resistance

At present, the main treatment method for HCC is radical surgery treatment. However, systemic therapy based on targeted medications is a crucial therapeutic alternative for patients who are not candidate for surgical treatment [3, 4, 32]. While the problem of targeted drug resistance raises great concern. The researches of resistance to sorafenib and lenvatinib have been extensively reported [33–35], but the mechanisms of resistance to regorafenib are rarely investigated. There have been existing studies demonstrated that HCC cells could mediate drug resistance by exosomes [18, 36]. Since we have proved that circDCAF8 can be transmitted between cells by exosomes and promote angiogenesis of HCC (Fig. 4), subsequent study was primarily focused on investigating whether integration of circDCAF8 into exosomes may confer regorafenib resistance.

**Fig. 8:**
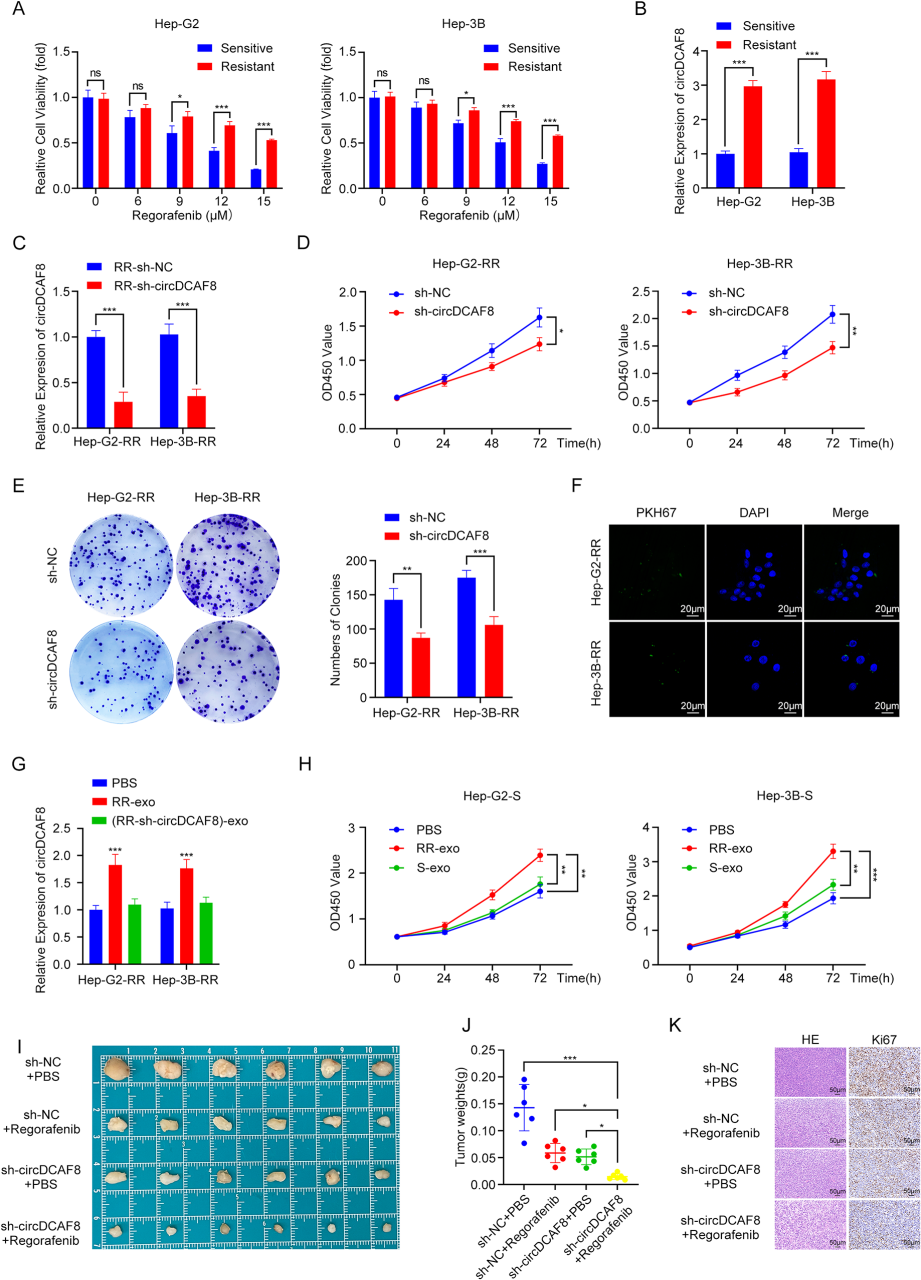
CircDCAF8 transmits regorafenib resistance by exosomes. **A** Cell viability of cells treated with different doses of regorafenib for 48 h. **B** Relative expression of circDCAF8 in regorafenib sensitive and resistant cells. **C** The knockdown efficiency of circCAF8 in regorafenib resistant cells were determined by qRT-PCR. **D, E** CCK8 and colony formation assays evaluated the proliferation of sh-circDCAF8 regorafenib resistant cells. **F** Exosomes secreted by regorafenib resistant cells were ingested by sensitive cells. Scale bar = 20 μm. **G** qRT-PCR was used to detect the relative expression of circDCAF8 in sensitive cells after incubation with exosomes from different sources. **H** CCK8 assay detected proliferation capacity in exosome-treated sensitive cells. **I** Regorafenib therapy or circDCAF8 knockdown could both inhibit the proliferation of the tumors formed in nude mice. **J** The weight of the subcutaneous tumor. **K** H&E and Ki67 staining of xenograft tumors. Scale bar = 50 μm. Data are presented as means ± SDs. In A-E, G, H, *n* = 3; in I-K, *n* = 6. (ns, not significant; *p < 0.05; **p < 0.01; ***p < 0.001)

Firstly, we established 2 cell lines that were resistant to regorafenib (Hep-G2-RR and Hep-3B-RR) by long-term exposure to low to high concentrations of regorafenib. The Fig. S5A displayed the IC50 values for the two regorafenib-resistant and regorafenib-sensitive cell lines (Hep-G2-S and Hep-3B-S). At different regorafenib concentrations, cell viability of regorafenib-resistant cells was notably increased compared to sensitive cells (Fig. 8A). As expected, the results of qRT-PCR analysis confirmed that Hep-G2 and Hep-3B cells with regorafenib resistance exerted higher circDCAF8 levels compared to sensitive cells (Fig. 8B). We then suppressed circDCAF8 in Hep-G2-RR and Hep-3B-RR cells to investigate the relationships between circDCAF8 and regorafenib resistance in HCC. The effectiveness of the knockdown was verified by qRT-PCR (Fig. 8C). As demonstrated by CCK8 and colony formation assays, the two regorafenib-resistant HCC cell lines’ regorafenib sensitivity was noticeably elevated upon circDCAF8 knockdown (Fig. 8D, E). Collectively, circDCAF8 is critical for maintaining regorafenib resistance. In addition, western blot assay revealed the expression of NAP1L1 and EMT-related proteins N-cadherin and Vimentin was significantly increased, whereas E-cadherin expression was decreased in regorafenib resistant cells compared with those in sensitive cells, indicating that circDCAF8 may promote regorafenib resistance in HCC by regulating NAP1L1 and EMT (Fig. S5B).

To verify whether exosomal circDCAF8 could mediate regorafenib resistance, we then isolated exosomes from the Hep-G2-RR and Hep-3B-RR cells. TEM, NTA and WB were performed same as above to identify exosomes isolated from the cells (Fig. S5C, D). Furthermore, qRT-PCR revealed that circDCAF8 was present in higher levels in exosomes isolated from Hep-G2-RR and Hep-3B-RR cells than in those from sensitive cells, indicating that exosomes could be able to transfer circDCAF8. (Fig. S5E).

After the exosomes isolated from Hep-G2-RR and Hep-3B-RR cells were characterized, they were labeled with PKH67 and incubated with sensitive cells for 24 h, respectively. Hep-G2 and Hep-3B sensitive cells showed green fluorescence signals under confocal laser microscopy after 24 h of co-cultivation (Fig. 8F), suggesting that the exosomes were successfully internalized. The exosomes of circDCAF8 knockdown regorafenib-resistant cells were extracted, identified and ingested in an identical manner. Next, we used qRT-PCR to ascertain cicrDCAF8 expression in sensitive cells. The findings showed that incubation with exosomes extracted from the regorafenib-resistant cells resulted in a higher amount of circDCAF8, however exosomes from regorafenib-resistant cells that had their circDCAF8 knockdown failed to increase the levels of circDCAF8 in sensitive cells (Fig. 8G). These aforementioned studies demonstrated that circDCAF8 could be transferred by exosomes from regorafenib-resistant cells to sensitive cells. Lastly, we investigated the possibility that circDCAF8 transported by exosomes could induce sensitive cells to become resistant to regorafenib. CCK8 assays demonstrated a substantial decrease in the cells’ sensitivity to regorafenib following incubation with exosomes extracted from regorafenib-resistant cell lines(Fig. 8H). The Colony formation assay revealed the same trend (Fig. S5F). Next, we made an effort to validate the function of circDCAF8 in regorafenib resistance in vivo. After creating the xenograft tumor model, it was shown that regorafenib therapy or circDCAF8 knockdown could both inhibit the proliferation of the tumor. However, the most notable suppression of tumor development was obtained with the combination of regorafenib therapy and circDCAF8 knockdown (Fig. 8I.J, Fig. S5G). IHC staining also revealed both circDCAF8 knockdown and regorafenib treatment reduced the level of Ki67, while the combination of the two decreased most (Fig. 8K). In conclusion, our research showed that circDCAF8 may be transferred by exosomes from regorafenib-resistant HCC cells to sensitive cells, conferring the sensitive cell lines a regorafenib resistant phenotype.

## Discussion

Even with various therapy advancements, HCC is still among the world’s most fatal cancers [1]. In addition to surgical resection, HCC molecular targeted drugs have obtained great success, but resistance is still the major influential factors of drug efficacy [3, 37, 38]. Plenty of previous studies have demonstrated that circRNAs have significant functions in the tumorigenesis and progression in human tumors [8, 9]. Numerous investigations have verified the function of circRNAs in the diagnosis and treatment of HCC [39, 40]. Dong, Z R et al. [41] proved circMEMO1 suppress HCC progression and increase sorafenib treatment sensitivity by modulating the promoter methylation and expression of TCF21. Dong, W et al. [42] reported circular RNA SCD-circRNA2 promotes the proliferation of HCC by interacting with the RNA-binding protein RBM3. To further explore the functional potential circRNAs involved in HCC carcinogenesis and progression, we analyzed the GSE94508 database. Based on strict inclusion criteria, circDCAF8 was identified and selected for further investigation. In this investigation, we discovered that circDCAF8 was substantially elevated in HCC tissues and was associated with the prognosis of HCC. In vitro experiments revealed that circDCAF8 knockdown suppressed cell proliferation, invasion, migration and EMT pathway, which were all improved by circDCAF8 overexpression. Furthermore, it was discovered through in vivo experiments that circDCAF8 stimulated HCC proliferation and metastasis. In consequence, it was confirmed that circDCAF8 had a carcinogenic effect in HCC and contributed to the occurrence and progression of HCC.

Tumor angiogenesis is regarded as one of the distinguishing characteristics of malignant tumors and is necessary for the sustained survival and development of tumor cells. It also plays a significant role in proliferation, invasion, and metastasis [23, 43]. Several studies on the effect of circRNAs in exosomes on HCC angiogenesis have been reported [44]. Huang, X Y et al. [25] have discovered that exosomal circRNA-100,338 enhanced angiogenesis to promote HCC metastasis. In our research, circDCAF8 was verified to be secreted via exosomes. After co-culture of exosomes with circDCAF8 knockdown or overexpression and HUVECs, exosome-delivered circDCAF8 were demonstrated to promote the proliferation, migration and tube formation of HUVECs. The above research suggested that upregulation of circDCAF8 in HCC promotes angiogenesis via exosomes.

Mechanistically, circRNAs have been shown in many studies to contribute to malignancies via the processes of miRNA sponges, including HCC [45]. Xu, L et al. [46] found circSETD3 acts as a sponge for miR-421 inhibiting HCC growth. Based on database prediction, the most promising downstream miRNA of circDCAF8 in HCC was found to be miR-217. We further confirmed that circDCAF8 and miR-217 interact in HCC by conducting RIP and dual luciferase reporter experiment. Our mechanism research revealed that miR-217 may have an interaction with NAP1L1. Nucleosome assembly protein 1-like 1 (NAP1L1) belongs to the human nucleosome assembly protein 1-like family(NAP1L1-6) [47], which plays a role in the assembly of nucleosomes, histone transport, histone efflux, transcriptional regulation, and cell cycle progression [48]. NAP1L1 and NAP1L4 are widely present in many human tissues, whereas others are mostly expressed in brain regions [49]. A number of studies have reported NAP1L1 is upregulated and has a carcinogenic function in various tumors such as breast cancer [50], pancreatic neuroendocrine neoplasm [51], colon cancer [52] and so on. In HCC, NAP1L1 has also been proven to facilitate tumor progression by activating multiple different signaling pathways [27, 53, 54]. Le Y et al. [55] discovered that NAP1L1 is associated with HCC doxorubicin chemotherapy resistance. This indicates us that NAP1L1 is a promising candidate for our study.

In our study, circDCAF8 positively regulated NAP1L1 expression in HCC cells, whereas miR-217 negatively regulated it. In rescue assays, the promotion of circDCAF8 overexpression on HCC progression could be inhibited by both miR-217 mimics and NAP1L1 inhibitor. According to all the information above, circDCAF8 promoted HCC progression through the miR-217/NAP1L1 axis.

Epithelial-Mesenchymal Transition (EMT) is a reversible process in which epithelial cells lose their own characteristics and become mesenchymal cells. This process can be activated by a variety of signals and regulatory networks of multiple transcription factors and processes including post-transcriptional and post-translational modifications as well as epigenetic modification [20]. In this process, tightly connected epithelial cells gain polarity while losing cell adhesion or homogeneous components, thereby increasing the strength and flexibility of the cytoskeleton and the ability to migrate and invade tissues [56]. During transformation, epithelial genes such as E-cadherin and ZO-1are lost in expression or function, whereas genes defining the mesenchymal phenotype such as Vimentin and N-cadherin are enhanced [57]. Activation of EMT plays an important role in tumor progression and is a crucial driver of tumor metastasis, because EMT can give tumor cells metastatic characteristics, enhance migration and invasion ability, and metastasis and colonization in distant organs [58]. In our study, knockdown of circDCAF8 upregulated the expression of E-cadherin and downregulated the expression of N-cadherin and Vimentin. After overexpression of circDCAF8, the expression of E-cadherin was downregulated, and the expression of N-cadherin and Vimentin was upregulated. Wound healing assay and transwell assay proved that overexpression of circDCAF8 promoted the migration and invasion ability of HCC cells. In vivo experiments also demonstrated that N-cadherin and Vimentin were highly expressed in tumor cells of circDCAF8 overexpression group, and upregulation of circDCAF8 promoted HCC lung metastasis. According to these aforementioned findings, circDCAF8 may active the EMT process to stimulate HCC invasion and metastasis. Mechanistically, circDCAF8 plays the cancer-promoting role through the miR-217/NAP1L1 axis. In rescue assays, the downregulation of E-cadherin and the upregulation of N-cadherin and Vimentin caused by circDCAF8 overexpression were reversed by miR-217 overexpression and NAP1L1 knockdown, indicating that circDCAF8 controls EMT process by regulating NAP1L1. To further investigate how NAP1L1 improves EMT in HCC, we focused on UBE2O, a downstream target of NAP1L1. Ubiquitin-conjugating enzyme E2O (UBE2O) is an E2 ubiquitin-conjugating enzyme that functions as a mixture of E2 and E3 enzymes and has both E2 and E3 activities. UBE2O has been found to be associated with the malignant progression of a variety of tumors, including HCC [59–61]. In breast cancer, UBE2O promoted EMT through the UBE2O/AMPKα2/mTORC1 axis [31], but the relationship between UBE2O and NAP1L1 and EMT in HCC has not been studied. After verifying the binding of NAP1L1 to UBE2O, we knocked down UBE2O in circDCAF8 overexpression and NAP1L1 overexpression Hep-3B cells to investigate the relationship between UBE2O and EMT. The results of western blot and transwell assay demonstrated that knockdown of UBE2O inhibited the migration, invasion and EMT of HCC cells, and overturned the effect of circDCAF8 and NAP1L1 overexpression on activating the EMT process. In conclusion, NAP1L1 can promote HCC EMT, migration and invasion by targeting UBE2O. Apart from facilitating invasion and metastasis, EMT has demonstrated the ability to enhance chemoresistance. For example, gemcitabine therapy for pancreatic ductal cancer can be more effective when EMT is inhibited [62]. In regorafenib resistant cell lines we induced, western blot assay revealed the expression of NAP1L1 and EMT-related proteins N-cadherin and Vimentin was significantly increased, whereas E-cadherin expression was decreased compared with those in sensitive cells, indicating that EMT process may promote regorafenib resistance in HCC.

The US Food and Drug Administration (FDA) authorized regorafenib, a multikinase inhibitor, in 2017 for the treatment of HCC patients who had previously received sorafenib treatment. Regorafenib is also approved for the second-line treatment of HCC in China [63, 64]. However, although regorafenib has a much richer set of targets, there are still drug resistance issues in clinical applications. Therefore, the mechanism of regorafenib resistance urgently needs to be elucidated. Numerous pieces of evidence have demonstrated the critical role circRNAs play in drug resistance. As an example, Xu, J et al. [33] found circRNA-SORE stabilizes YBX1 to mediate sorafenib resistance in HCC. CircMED27 reported by Zhang, P et al. [65] enhance lenvatinib resistance in HCC by sponging miR-655-3p to regulate USP28 expression. Additionally, circRNAs have also been reported to be packaged into exosomes and associated with HCC drug resistance. Hu, Z et al. [36] demonstrated circCCAR1 derived from exosomes induced dysfunction of CD8^+^ T-cells and resistance to anti-PD1 therapy in HCC.

Since circDCAF8 has been demonstrated in our earlier studies to be secreted by exosomes and to stimulate angiogenesis, we postulated that circDCAF8 may impact HCC’s chemosensitivity through exosomes. As we assumed, circDCAF8 was expressed more in regorafenib-resistant cell lines than in sensitive cells, and circDCAF8 knockdown caused regorafenib-resistant cells more sensitive, suggesting a strong relationship between circDCAF8 and regorafenib resistance. Furthermore, we demonstrated exosomes released from regorafenib resistant cells can mediate the transfer of circDCAF8 and the transmission of regorafenib resistance, which provided a new potential target for overcoming regorafenib resistance.

## Conclusion

To sum up, our research demonstrated that circDCAF8 enhanced the development of HCC by targeting on the miR-217/NAP1L1 axis. In addition, circDCAF8 was confirmed to be secreted from HCC cells via exosomes and involved in angiogenesis and transmission of regorafenib resistance. All things considered, our research points out circDCAF8 is a possible therapeutic target for HCC patients and provide a meaningful revelation for reversing regorafenib resistance.

### Electronic supplementary material

Below is the link to the electronic supplementary material.

Supplementary Material 1

Supplementary Material 2

## Data Availability

The circRNA sequencing database GSE94508( https://www.ncbi.nlm.nih.gov/geo/query/acc.cgi?acc=GSE94508 ) was obtained from Gene Expression Omnibus (GEO). All data generated or analyzed during this study are contained in this paper or supplementary materials. Processed data are available from the corresponding author upon reasonable request.

## Abbreviations

HCC: Hepatocellular Carcinoma
circRNAs: Circular RNAs
miRNA: MicroRNA
shRNA: Small hairpin RNA
qRT-PCR: Quantitative real-time polymerase chain reaction
RIP: RNA-binding protein immunoprecipitation
EMT: Epithelial-mesenchymal transformation
NAP1L1: Nucleosome assembly protein 1-like 1
UBE2O: Ubiquitin-conjugating enzyme E2O
