# Supplementary material for: CircDCAF8 promotes the progression of hepatocellular carcinoma through miR-217/NAP1L1 Axis, and induces angiogenesis and regorafenib resistance via exosome-mediated transfer
Source: J Transl Med. 2024 May 30;22:517. doi: 10.1186/s12967-024-05233-4 (PMC11137954; doi:10.1186/s12967-024-05233-4)
Supplement: Supplementary file 1 — Supplementary Material 1 [file 12967_2024_5233_MOESM1_ESM.docx]

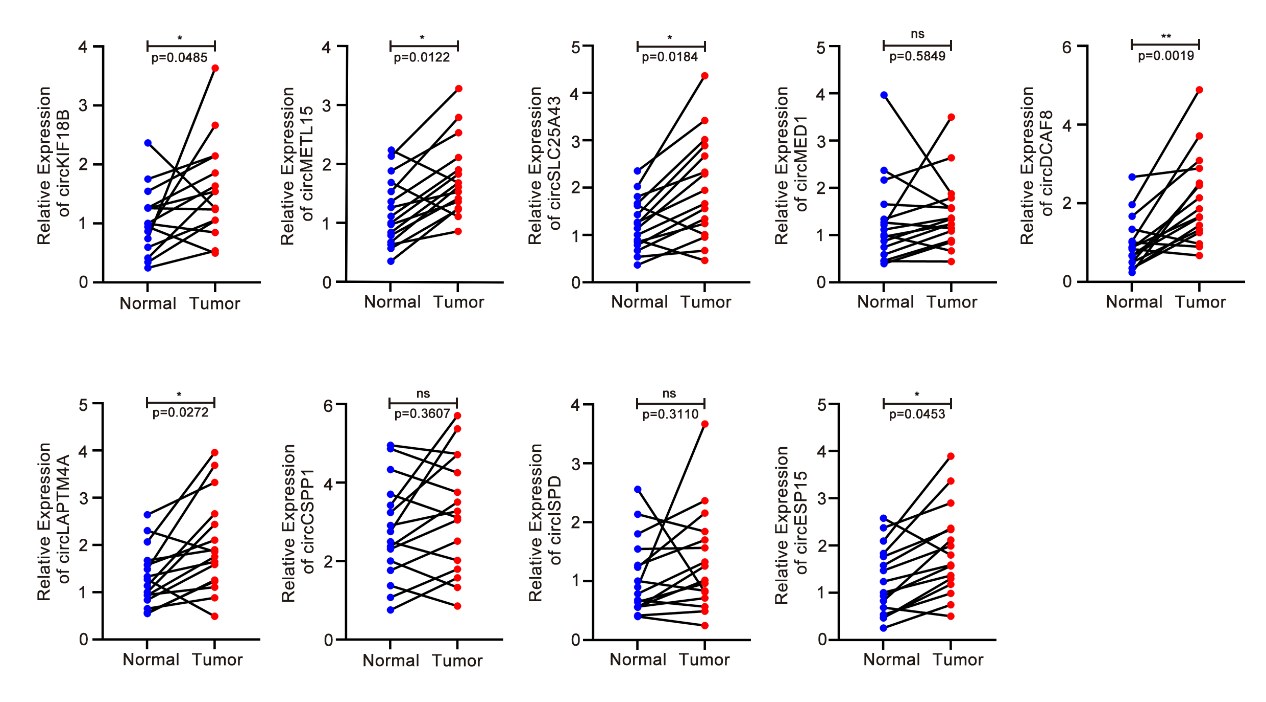


**Figure.S1** Relative expression of 9 candidate circRNAs in human HCC tissues and paired adjacent nontumor tissues of 16 patients was determined by qRT-PCR. Data are representative of three independent experiments and are presented as means ± SDs. (*p<0.05; **p<0.01; ***p<0.001).


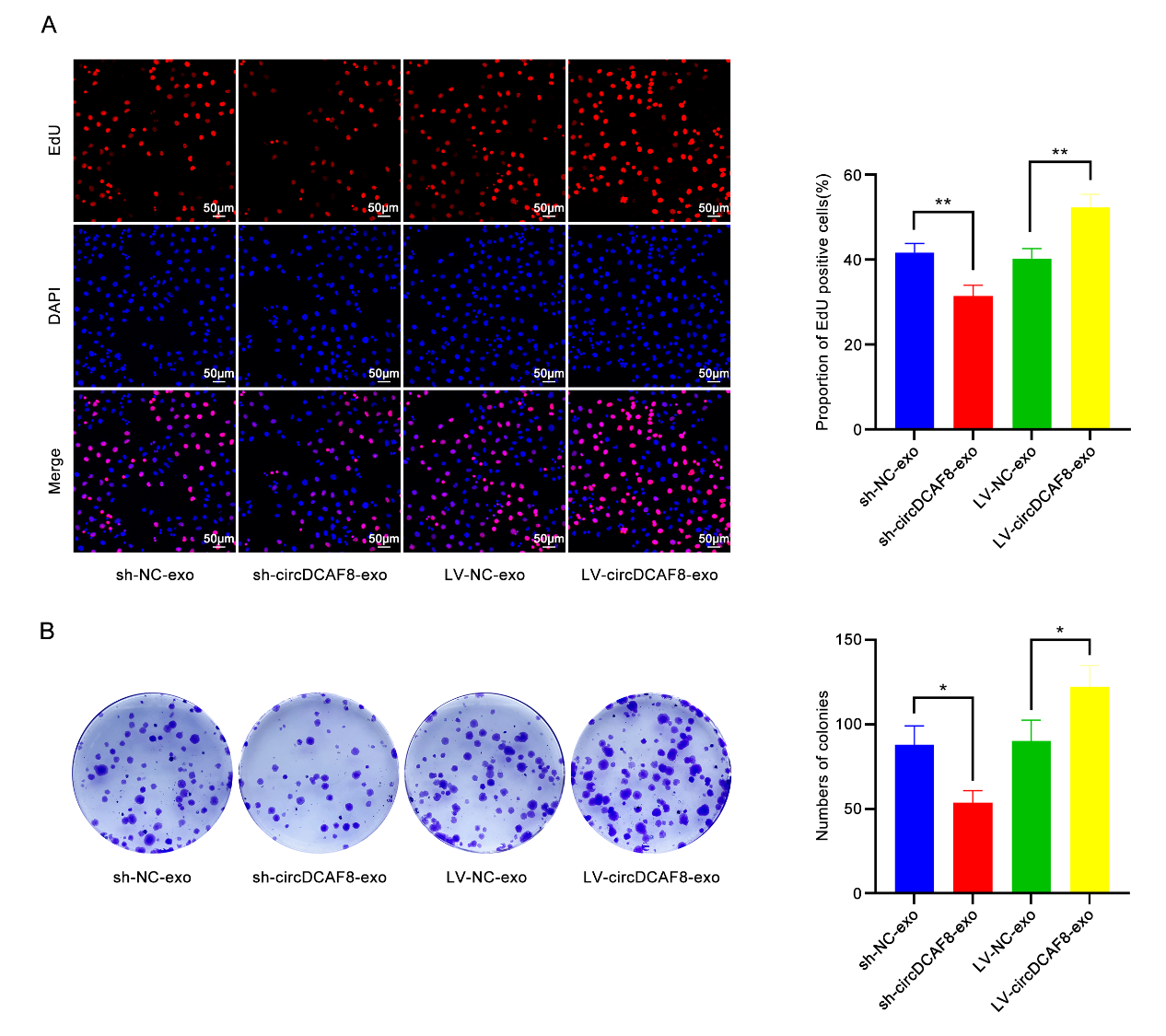


**Figure.S2** **A** EdU assay of HUVECs ingested exosomes from circDCAF8 knockdown or overexpression cells, Scale bar=50μm. **B** Colony formation assay of HUVECs ingested exosomes from circDCAF8 knockdown or overexpression cells. Data are representative of three independent experiments and are presented as means ± SDs. (*p<0.05; **p<0.01; ***p<0.001).

**
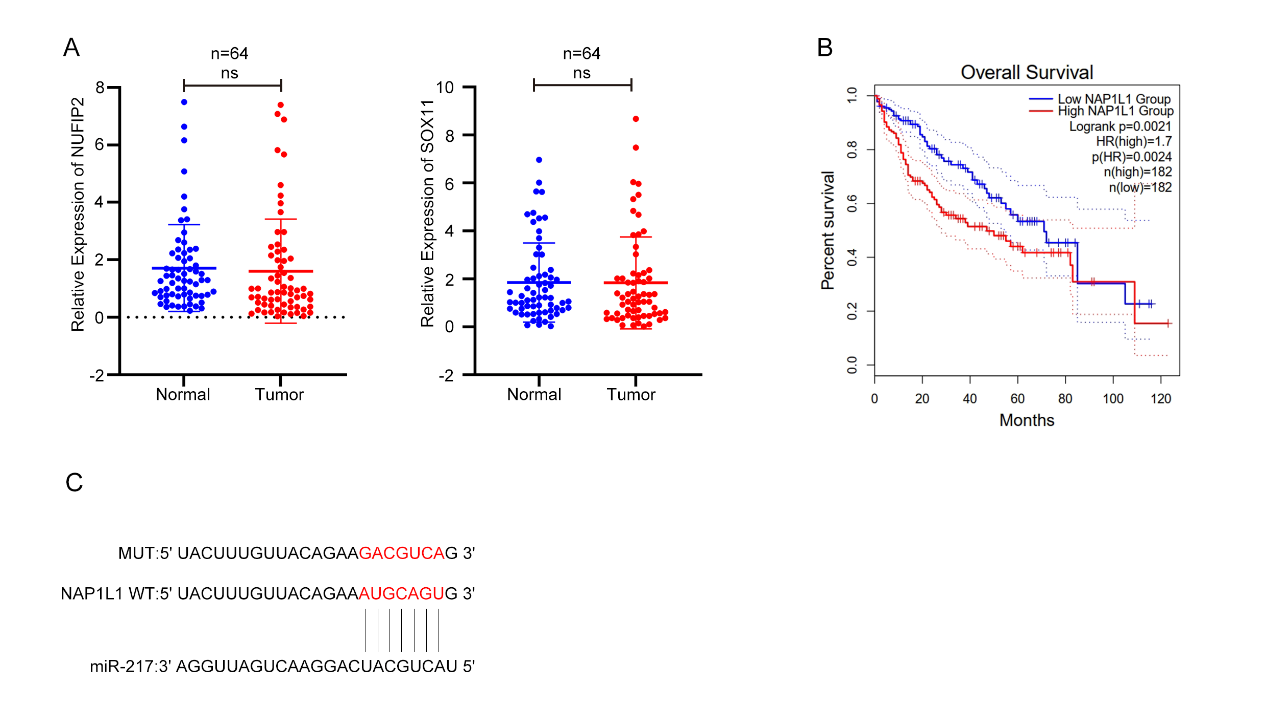
**

**Figure.S3** **A** Relative expression of NUFIP2 and SOX11 in human HCC tissues and paired adjacent nontumor tissues of 64 patients was determined by qRT-PCR. **B** Overall survival of HCC patients with high versus low NAP1L1 levels. **C** A schematic of wild-type (WT) and mutant (MUT) NAP1L1 luciferase reporter vectors. Data are representative of three independent experiments and are presented as means ± SDs. (*p<0.05; **p<0.01; ***p<0.001).


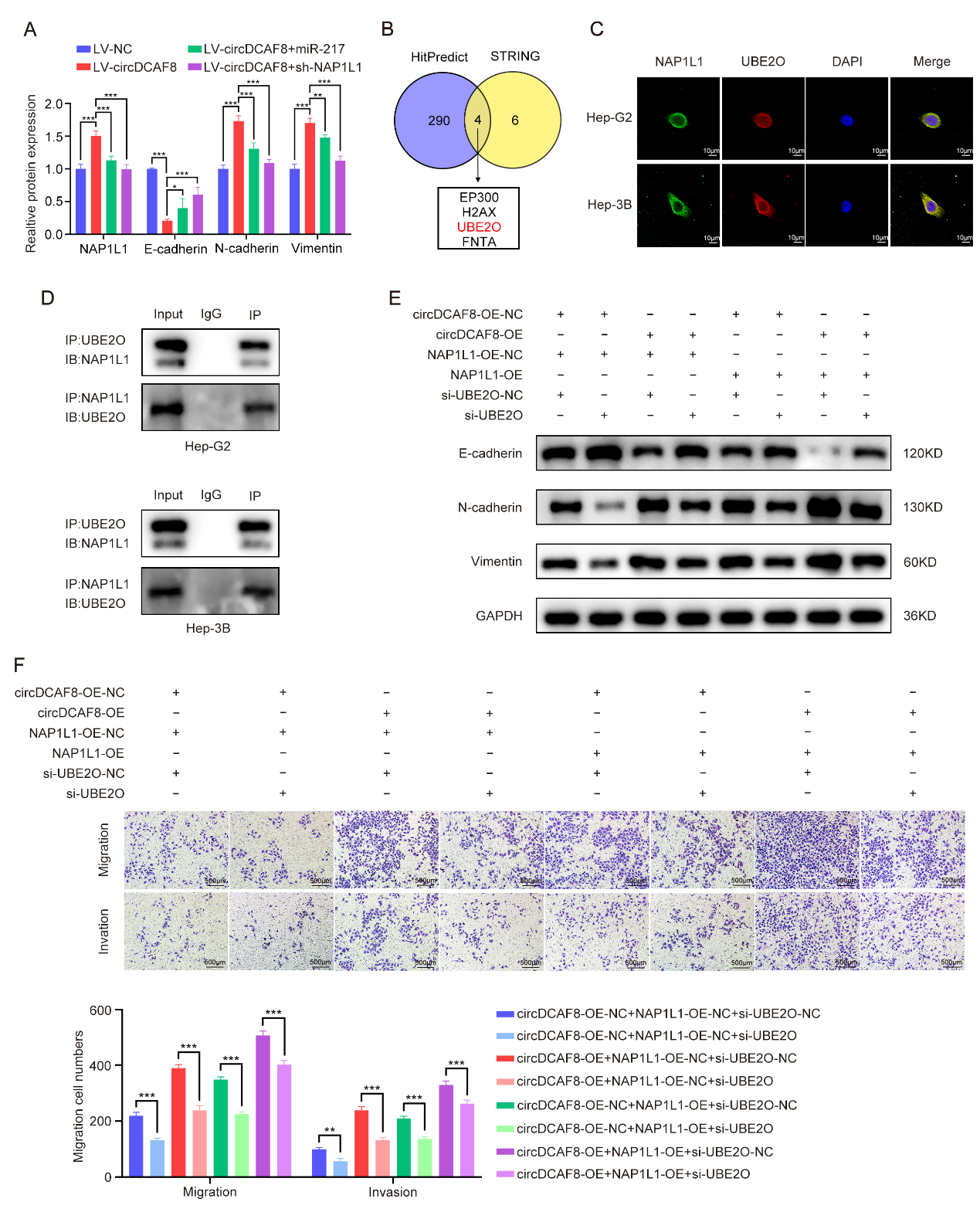


**Fig.S4 A** Quantification of NAP1L1, E-cadherin, N-cadherin and Vimentin protein levels of Fig.7F. **B** Downstream proteins of NAP1L1 predicted by HitpPedict and STRING databases. **C** The co-localization of NAP1L1 and UBE2O detected by immunofluorescence assays, scale bar=10μm. **D** The binding of NAP1L1 to UBE2O was confirmed by co-IP with NAP1L1 or UBE2O antibody. **E** Western blot detected the expression of EMT-related proteins in Hep-3B cells transfected with LV-circDCAF8, LV-NAP1L1 or si-UBE2O. **F** Transwell assay measured migration and invasion ability in Hep-3B cells transfected with LV-circDCAF8, LV-NAP1L1 or si-UBE2O. Scale bar=500μm. Data are representative of three independent experiments and are presented as means ± SDs. (*p<0.05; **p<0.01; ***p<0.001).


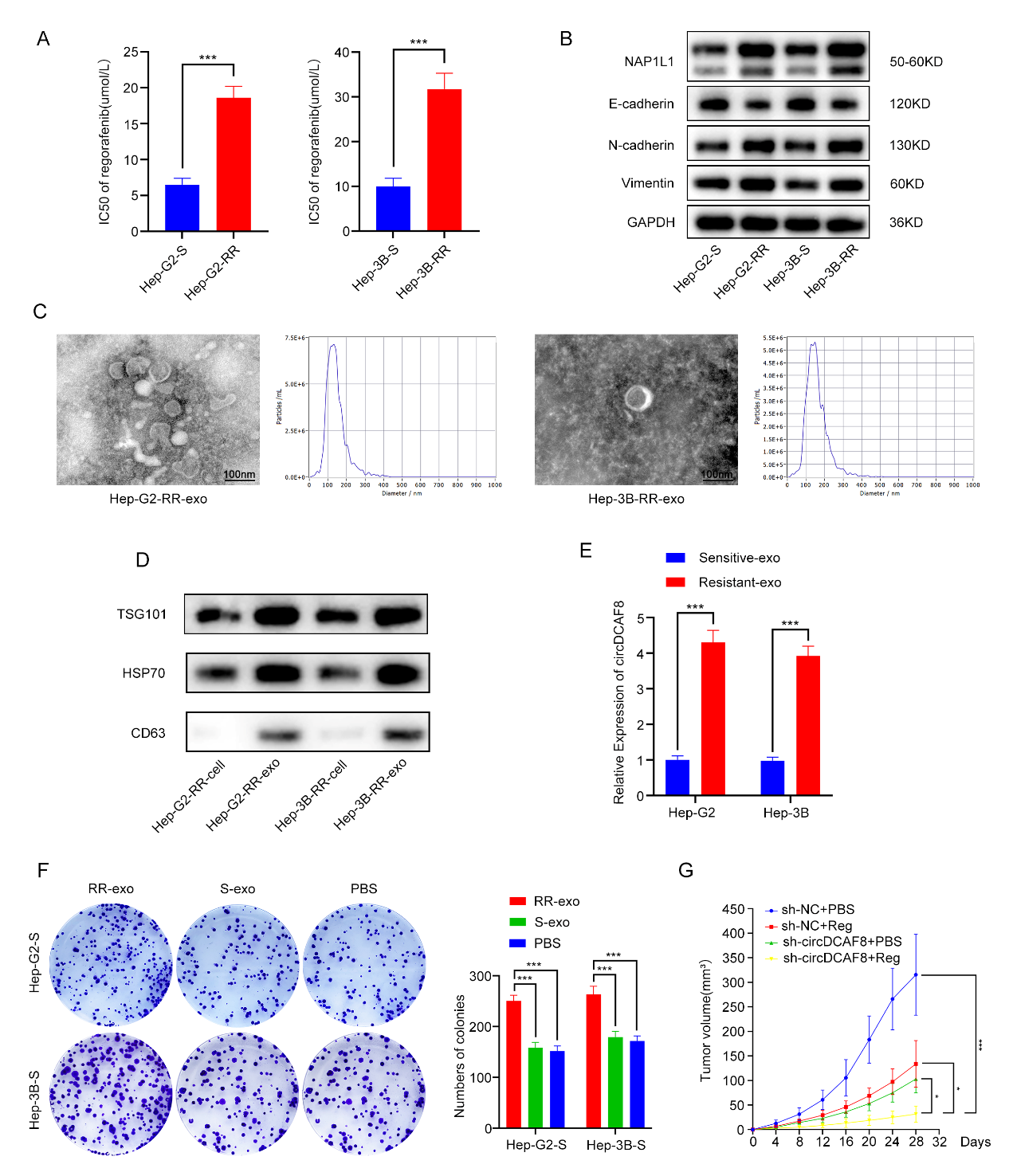


**Figure.S5** **A** IC50 of regorafenib resistant cell lines and sensitive cells. **B** Western blot detected the expression of NAP1L1 and EMT-related proteins in regorafenib sensitive and resistant HCC cells. **C** TEM and NTA of exosomes isolated from Hep-G2 and Hep-3B. Scale bar=100nm. **D** Exosomal protein positive markers (Tsg101, HSP70 and CD63) were detected by western blot from purified exosomes and exosome-depleted cell extracts. **E** Relative expression of circDCAF8 in exosomes extracted from regorafenib resistant cells compared to sensitive cells. **F** Colony formation assay of exosome-treated sensitive cells. **G** The volume of the subcutaneous tumor. Data are representative of three independent experiments (in G, n=6) and are presented as means ± SDs. (*p<0.05; **p<0.01; ***p<0.001).
