# Supplementary material for: CircDCAF8 promotes the progression of hepatocellular carcinoma through miR-217/NAP1L1 Axis, and induces angiogenesis and regorafenib resistance via exosome-mediated transfer
Source: J Transl Med. 2024 May 30;22:517. doi: 10.1186/s12967-024-05233-4 (PMC11137954; doi:10.1186/s12967-024-05233-4)
Supplement: Supplementary file 2 — Supplementary Material 2 [file 12967_2024_5233_MOESM2_ESM.docx]

**Table S1. ShRNA/siRNA sequence used in this study.**

| sh-RNA/si-RNA | Target sequence (5’-3’) |
| --- | --- |
| sh-circ DCAF8 | GCACCAAACTGACCTGTTTGA |
| sh-NAP1L1  si-UBE2O | GGGTTATCAGCTCTTTGAAGA  GCAGGUUGUAGAGUUGAAATT |

**Table S2. Full sequence information of hsa_circ_0014879**

| > hsa_circ_0014879 |
| --- |
| TCCTTCAGTGAATCTACAGACCTATTTTCTCAGGAGCTCAGCCTGGCCTTACTTCAGTGATAAAAGGAGGAAAGGCTGGCTACAGCAAACATCATTCAAGATGTCCAGCAAAGGGAGCAGCACAGATGGCAGAACAGACTTAGCTAATGGAAGCCTGTCTAGCAGTCCAGAGGAGATGTCTGGAGCTGAAGAGGGGAGGGAGACATCCTCAGGCATTGAAGTGGAGGCCTCAGACCTGAGTTTGAGCTTGACTGGGGATGATGGTGGCCCCAACCGCACCAGCACAGAAAGTCGAGGCACAGACACAGAGAGCTCAGGTGAAGATAAGGACTCTGACAGCATGGAGGACACTGGTCATTACTCCATTAATGATGAAAATCGAGTCCATGACCGCTCAGAGGAAGAGGAAGAGGAGGAAGAAGAGGAGGAAGAAGAGCAGCCTCGGCGCCGTGTACAGCGCAAGCGGGCTAACCGTGACCAGGACTCATCAGATGATGAGCGGGCCCTAGAGGACTGGGTGTCCTCAGAAACATCAGCTCTACCCCGACCTCGCTGGCAAGCCCTCCCTGCCCTTCGGGAGCGGGAGCTGGGTTCAAGTGCCCGCTTTGTCTATGAGGCCTGTGGGGCAAGAGTCTTTGTGCAGCGTTTCCGCCTGCAGCATGGGCTTGAGGGCCATACTGGTTGTGTCAATACCCTGCACTTTAACCAGCGCGGCACCTGGCTGGCCAGTGGCAGCGATGACCTGAAGGTGGTGGTGTGGGATTGGGTACGGCGGCAGCCAGTACTGGACTTTGAGAGTGGCCACAAAAGTAATGTGTTCCAGGCCAAGTTTCTTCCTAACAGTGGTGATTCTACTCTGGCCATGTGTGCCCGTGACGGGCAGGTTCGAGTAGCAGAACTGTCTGCCACACAGTGTTGCAAGAATACAAAACGTGTGGCCCAGCACAAGGGAGCGTCCCACAAGTTGGCACTGGAACCAGACTCTCCCTGTACGTTCTTATCTGCAGGTGAAGATGCAGTTGTTTTCACCATTGACCTGAGACAAGACCGCCCAGCGTC |

**Table S3. Primer sequences used in this study.**

| Gene | | Sequence (5’-3’) |
| --- | --- | --- |
| DCAF8 | F | CAGATGGCAGAACAGACTTAGC |
|  | R | CTCCACTTCAATGCCTGAGGA |
| CircDCAF8-divergent | F | ACCAGACTCTCCCTGTACGT |
|  | R | TGTAGATTCACTGAAGGAGACGC |
| CircDCAF8-convergent | F | CTGAAGAGGGGAGGGAGACA |
|  | R | CTGTGTCTGTGCCTCGACTT |
| GAPDH | F | GAACGGGAAGCTCACTGG |
|  | R | GCCTGCTTCACCACCTTCT |
| GAPDH-divergent | F | GAAGACTGTGGATGGCCCCT |
|  | R | CAAATGAGCCCCAGCCTTCT |
| MiR-217 | F | CGCGTACTGCATCAGGAACTG |
|  | R | AGTGCAGGGTCCGAGGTATT |
| NAP1L1 | F | ATGGCAGACATTGACAAC |
|  | R | TCACTGCTGCTTGCACTCG |

**Table S4. Antibodies used in this study**

| Antibody | Source |
| --- | --- |
| Anti-E-cadherin antibody | Proteintech |
| Anti-N-cadherin antibody | Proteintech |
| Anti-Vimentin antibody | Proteintech |
| Anti-TSG101 antibody | Proteintech |
| Anti-HSP70 antibody | Proteintech |
| Anti-CD63 antibody  Anti-NAP1L1 antibody  Anti-UBE2O antibody | Proteintech  Proteintech  Proteintech |
| Anti-GAPDH antibody  Anti-Ki67 antibody | Proteintech  Servicebio |
| Secondary antibody | Proteintech |
| Anti-AGO2 antibody | Cell Signaling Technology |
| Anti-IgG antibody | Cell Signaling Technology |

**Table S5. The top 13 upregulated circRNAs**

| GeneID | circBase ID | Symbol | Named by us  (circ+symbol) |
| --- | --- | --- | --- |
| chr1:160293220-160302347 | hsa_circ_0008661 | COPA | circCOPA |
| chr19:39090564-39090793 | hsa_circ_0050867 | MAP4K1 | circMAP4K1 |
| chr16:74670243-74671868 | hsa_circ_0004519 | RFWD3 | circRFWD3 |
| chr17:43012303-43012398 | hsa_circ_0000775 | KIF18B | circKIF18B |
| chr11:28250417-28255132 | hsa_circ_0000282 | METTL15 | circMETTL15 |
| chrX:118544152-118544325 | hsa_circ_0006789 | SLC25A43 | circSLC25A43 |
| chr17:37583953-37584043 | hsa_circ_0043438 | MED1 | circMED1 |
| chr1:160206924-160231148 | hsa_circ_0014879 | DCAF8 | circDCAF8 |
| chr2:20240809-20240905 | hsa_circ_0000981 | LAPTM4A | circLAPTM4A |
| chr8:68018139-68028357 | hsa_circ_0001806 | CSPP1 | circCSPP1 |
| chr4:57976053-57976143 | hsa_circ_0001416 | LOC255130 | circLOC255130 |
| chr7:16298014-16317851 | hsa_circ_0079480 | ISPD | circISPD |
| chr1:51829537-51831701 | hsa_circ_0012417 | EPS15 | circEPS15 |
